# MLN0128, a novel mTOR kinase inhibitor, disrupts survival signaling and triggers apoptosis in AML and AML stem/ progenitor cells

**DOI:** 10.18632/oncotarget.10397

**Published:** 2016-07-04

**Authors:** Zhihong Zeng, Rui-Yu Wang, Yi Hua Qiu, Duncan H. Mak, Kevin Coombes, Suk Young Yoo, Qi Zhang, Katti Jessen, Yi Liu, Christian Rommel, David A. Fruman, Hagop M. Kantarjian, Steven M. Kornblau, Michael Andreeff, Marina Konopleva

**Affiliations:** ^1^ Section of Molecular Hematology and Therapy, Department of Leukemia, The University of Texas MD Anderson Cancer Center, Houston, TX, USA; ^2^ Department of Bioinformatics and Computational Biology, The University of Texas MD Anderson Cancer Center, Houston, TX, USA; ^3^ Department of Biomedical Informatics, Ohio State University College of Medicine, Columbus, OH, USA; ^4^ Oncology-Rinat Research & Development, San Diego, CA, USA; ^5^ Wellspring Bioscience, San Diego, CA, USA; ^6^ Roche Innovation Center Basel, Basel, Switzerland; ^7^ Institute for Immunology, and Department of Molecular Biology and Biochemistry, University of California-Irvine, Irvine, CA, USA

**Keywords:** mTOR, AML, stem cells, CyTOF, therapy

## Abstract

mTOR activation leads to enhanced survival signaling in acute myeloid leukemia (AML) cells. The active-site mTOR inhibitors (asTORi) represent a promising new approach to targeting mTOR in AKT/mTOR signaling. MLN0128 is an orally-administered, second-generation asTORi, currently in clinical development. We examined the anti-leukemic effects and the mechanisms of action of MLN0128 in AML cell lines and primary samples, with a particular focus on its effect in AML stem/progenitor cells. MLN0128 inhibited cell proliferation and induced apoptosis in AML by attenuating the activity of mTOR complex 1 and 2. Using time-of-flight mass cytometry, we demonstrated that MLN0128 selectively targeted and functionally inhibited AML stem/progenitor cells with high AKT/mTOR signaling activity. Using the reverse-phase protein array technique, we measured expression and phosphorylation changes in response to MLN0128 in 151 proteins from 24 primary AML samples and identified several pro-survival pathways that antagonize MLN0128-induced cellular stress. A combined blockade of AKT/mTOR signaling and these pro-survival pathways facilitated AML cell killing. Our findings provide a rationale for the clinical use of MLN0128 to target AML and AML stem/progenitor cells, and support the use of combinatorial multi-targeted approaches in AML therapy.

## INTRODUCTION

The AKT/mTOR signaling pathway regulates cellular growth, survival, and proliferation [1, 2]. Dysregulation of this pathway has been observed in acute myeloid leukemia (AML), and is a key factor that attenuates the response of AML to conventional chemotherapy and contributes to drug resistance and AML relapse [3, 4]. Hyper-activated mTOR promotes cellular biosynthetic processes that are necessary for AML cell division and survival [5]. Therefore, targeting mTOR in AKT/mTOR signaling holds promise for AML therapy [6].

mTOR acts in two distinct complexes, mTOR complex 1 (mTORC1) and mTOR complex 2 (mTORC2). mTORC1 promotes protein translation and synthesis by phosphorylation of the substrates 4EBP1 and S6 kinase; mTORC2 controls cell survival and proliferation through downstream activation of AKT and AGC protein kinase [2, 7]. The classic mTOR inhibitor, rapamycin, and its analogues bind to an allosteric site in mTORC1 reducing mTORC1's activity on selected substrates [8]. These inhibitors have minimal effect on mTORC2 in most cancer cell types [9, 10]. The newer ATP-competitive mTOR inhibitors suppress phosphorylation of all mTORC1 and mTORC2 substrates. These active-site mTOR inhibitors (asTORi) are more effective than classic mTOR inhibitors in blocking protein synthesis [11, 12]. The first- and second- generation asTORi PP242 and MLN0128 (formerly known as INK128) demonstrated potent antitumor activities against various malignances in preclinical studies [13–19]. MLN0128 is an orally-administered asTORi, which is currently being investigated in phase I and II trials as a monotherapy or in combination with other therapeutic agents against advanced cancer (www.clinicalTrials.gov) [20–22].

Limited studies have been done to investigate the effects of mTORC1/C2 inhibition in AML [14, 23], particularly, in AML stem/progenitor cells, often called leukemic stem cells, constituting a small population of leukemic cells capable of self-renewal that contributes to residual disease [24]. Recent findings indicate that mTOR inhibition activated compensatory signaling through negative feedback from both mTORC1/C2 [25, 26]. mTOR inhibitors are most effective against cancer cells when used in combination with other therapies [13, 18]. However, until now, no thorough studies have been done to determine compensatory pathways triggered by mTOR inhibition in AML. Identifying druggable targets in these pathways, and knowing the effects of their blockade during mTOR inhibition, is critical to prevent drug resistance and improve the therapeutic efficacy of AML.

Several high-throughput technologies, such as mass cytometry time of flight (CyTOF) [27] and reverse-phase protein array (RPPA) [28] have been developed to advance studies of cellular biology at the single-cell level and to investigate intracellular pathway at the signaling network level. In this study we utilized CyTOF to identify AML stem/progenitor cells, and to determine their response to MLN0128. We applied RPPA to investigate signaling network alterations in primary AML blasts upon mTORC1/C2 inhibition. We demonstrated the anti-leukemic effects and the mechanisms of actions of MLN0128 in AML and AML stem/progenitor cells, and identified cellular survival mechanisms in response to MLN0128. We showed that combined blockade of AKT/mTOR signaling and druggable pro-survival targets facilitated AML cell killing.

## RESULTS

### MLN0128 inhibits cell growth and induces apoptosis in AML

**Figure 1:**
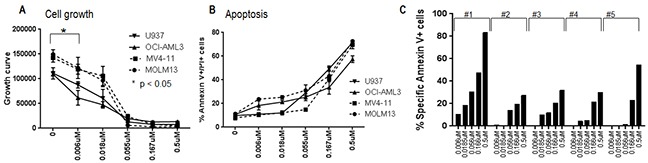
Anti-leukemic effect of MLN0128 in AML AML cell lines **A, B.** and AML progenitor cells **C.** were treated with different concentrations of MLN0128 for 72 hours. Growth inhibition of cell lines was measured by Vi-Cell XR cell viability analyzer. Apoptosis induction of cell lines and primary progenitor cells were measured by flow cytometry. Specific apoptosis was calculated as described in the Materials and Methods. Clinical information on primary AML samples is included in the Supplementary Table S1 apoptosis section. *: Cell growth was significantly inhibited at the lowest concentration of MLN0128 on all cell lines examined (Student *t*-test, p < 0.05).

The anti-leukemic efficacy of MLN0128 was examined in four AML cell lines: FLT3-ITD-mutated MOLM13 and MV4-11 cells; NPM1 and N-Ras-mutated OCI-AML3 cells; and in PTEN-null U937 cells. In a dose-dependent fashion, MLN0128 caused growth inhibition at low nanomolar concentrations, and induced apoptosis at higher concentrations (Figure 1A, B). A similar effect with apoptosis induction was observed in primary AML CD34+ progenitor cells with or without FLT3-mutations (Figure 1C). MLN0128 demonstrated a much higher anti-leukemic efficacy in primary AML than rapamycin (Supplementary Figure S5). Together, these results indicate that MLN0128 is a potent mTORC1/C2 kinase inhibitor that affects growth and survival of AML cells.

### MLN0128 blocks the activation of mTORC1/C2 downstream targets in AML

**Figure 2:**
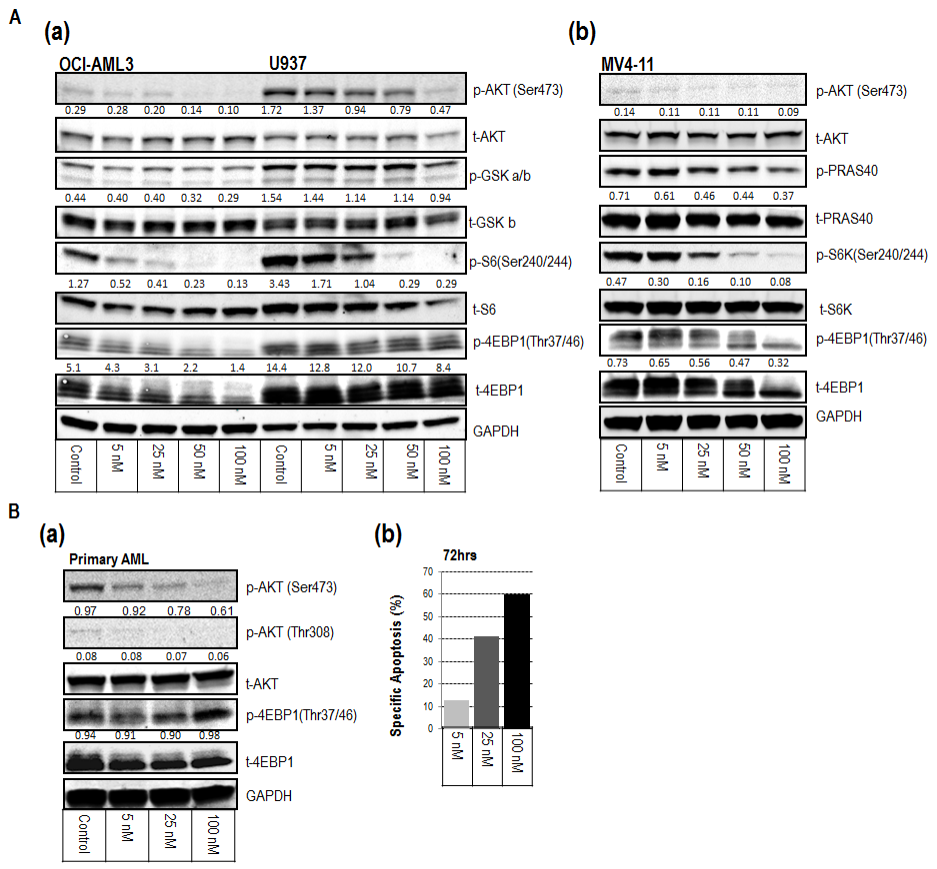
Inhibitory mechanisms of MLN0128 on AML cell lines (A) and on a representative primary AML sample (B) **A (a-b).** and **B (a).** Western blot revealed the inhibition of downstream targets of mTORC1 and mTORC2 at the indicated concentrations of MLN0128 for 1 hour. Protein intensity was quantified. The intensity of phosphorylated protein was calculated using the formula: p-protein/t-protein/GAPDH x 100, except for p-4EBP1 in (a), which used p-protein/GAPDH x 100. Results are displayed under the blot of phosphorylated protein. p-protein = phosphorylated protein; t-protein = total protein. **B (b).** Bar graph displays the specific apoptosis induction of the same sample at 72 hours.

To confirm the on-target molecular mechanisms of MLN0128, we assessed inhibition of AKT/mTOR signaling in three AML cell lines (OCI-AML3, U937, and MV4-11) and primary AML samples by Western blot analysis. MLN0128 attenuated the activity of both mTORC1 and mTORC2 at nanomolar concentrations. MLN0128 inhibited AKT phosphorylation at the mTORC2 site Ser473 and reduced phosphorylation of AKT substrates PRAS40 and GSK. It also blocked phosphorylation of mTORC1 substrates S6 (Ser240/244) and the rapamycin-resistant site on p-4EBP1 (Thr37/46) (Figure 2A a-b and 2B a). Apoptosis induction as a result of mTORC1/C2 inhibition is shown for a representative primary AML sample (Figure 2B b).

### MLN0128 inhibits AKT/mTOR signaling in AML stem/progenitor cells

MLN0128 induced apoptosis in AML CD34+ cells, prompting us to investigate its effects in AML stem/progenitor subpopulations defined by CyTOF, a novel technology that incorporates cytometry and mass spectrometry to measure multiple parameters without spectral overlap. Analysis of the CyTOF data by the analytic tools Spanning-Tree Progression of Density-Normalized Events (SPADE) [29] and visualization t-Distribution Stochastic Neighbor Embedding (viSNE) [30], allows us to identify and characterize signaling patterns of rare and heterogeneous population of leukemic stem cells. We composed a CyTOF antibody panel that recognizes stem cell surface markers CD34, CD123 [31], CD133 [32], CD47 [33], and CD99 [34]; chemokine and adhesion molecules CD44, CD49d, and CXCR4; and intracellular molecules p-AKT, p-mTOR, p-S6, p-4EBP1, p-ERK, p-STAT3 and p-STAT5 (Supplementary Table S3).

**Figure 3:**
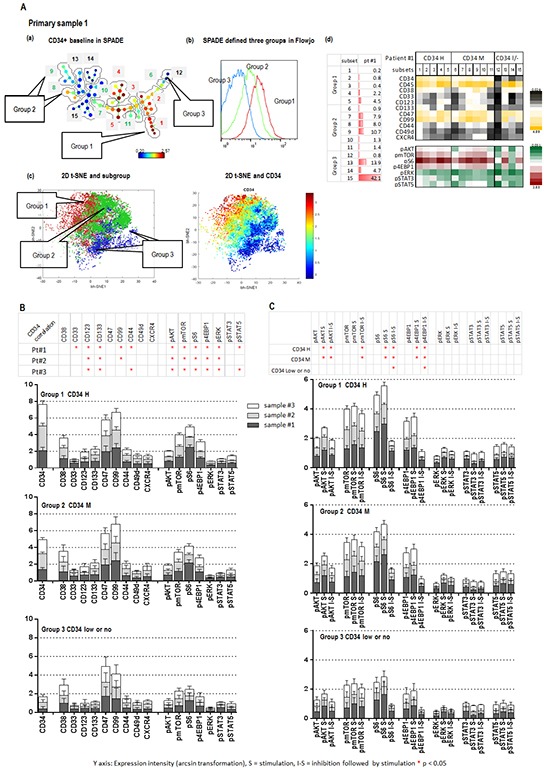
CyTOF identifies Inhibitory effect of MLN0128 on AML stem/progenitor cells **A (a).** SPADE displays patient #1 CD34 baseline expression. Subsets containing the similar intensity of CD34 were grouped as indicated (groups 1, 2 and 3). The defined groups were confirmed by a FlowJo histogram graph **A (b).** and a viSNE 2-D plot **A (c). (A (d) left):** cell frequency of defined subsets. **(A (d) right):** baseline expressions of surface and intracellular molecules of defined subsets of three groups. **B.** The expression intensities of surface and intracellular molecules in subsets of each group (groups 1, 2, and 3) of three samples (1, 2, and 3) are displayed in stack bar graphs. The *y*-axis shows: expression intensity (arcsin transformation). Table on the top summarizes the correlation between CD34 and surface and intracellular molecules. An asterisk indicates a significant correlation. **C.** Effect of SCF (S = Stimulation) and MLN0128 (I = Inhibition) on defined subsets of three groups in three samples. The expression intensity of intracellular molecules before and after SCF and that of MLN0128 followed by SCF (I-S) in the defined subsets of each group of three samples are displayed in stack bar graphs. Significant stimulation (before and after SCF) and inhibition (SCF with or without MLN0128) of intracellular molecules in each CD34 expression group of three samples are summarized in the table on top of the stack bar graphs. *: significance (p < 0.05).

SPADE analysis of 3 AML samples identified 15 unique subpopulations (subsets) with various cell frequencies and differential expression of surface and intracellular markers, as summarized in the table and heatmap (Figure 3A a and 3A d, sample #1; Supplementary Figure S1A and S1B, a and d samples #2 and #3; Supplementary Table S1 CyTOF section AML samples #1, #2 and #3). Fifteen subsets of each sample were divided into three groups based on CD34 expression: group 1, CD34 medium to high (5 subsets); group 2, positive to medium (6 subsets); group 3, low or no expression (4 subsets) (Figure 3A a; Supplementary Figure S1A and S1B, a). These three groups were cross-validated by viSNE and FlowJo (Figure 3A b and 3A c; Supplementary Figure S1A and S1B, b and c). CD34 expression positively correlated with the expression of stem cell surface molecules CD123 and CD133 and the intracellular baseline level of p-AKT, p-mTOR, p-S6, p-4EBP1 and p-ERK in all three groups of the three samples tested (Figure 3B). Positive correlations between CD34 and CD99 were observed in samples 1 and 2; between CD34 and the adhesion molecule CD44 and the intracellular p-STAT5 were observed in samples 1 and 3. Stem cell factor (SCF) significantly increased the expression level of p-AKT, p-S6 and p-4EBP1 in subsets of groups 1 and 2 with positive to high CD34, but showed less effect on the same molecules in the subsets of group 3 with low or no expression of CD34 (Figure 3C). Pre-treatment with MLN0128 effectively blocked SCF-induced upregulation of p-AKT, p-S6, p-4EBP1 and p-mTOR in subsets of groups 1 and 2, and of p-S6 and p-4EBP1 in subsets of group 3 (Figure 3C; Supplementary Figure S2). Together, our data support the notion that AML stem/progenitor subsets are heterogeneous; that subsets with high expression of CD34 co-express multiple stem cell surface markers and had high basal intracellular signaling; and AKT/mTOR signaling in these subsets was sensitive to SCF stimulation and MLN0128 inhibition.

### MLN0128 inhibits the function of AML-derived xenograft leukemic cells carrying FLT3 mutation

**Figure 4:**
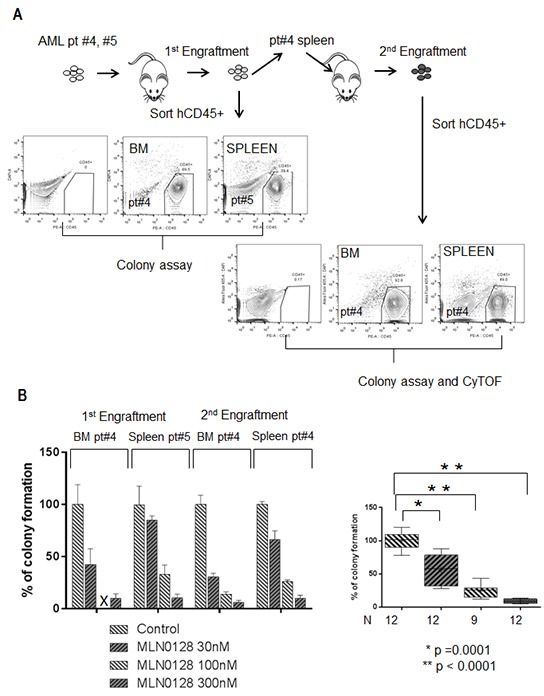
MLN0128 inhibits the function of AML-derived xenograft leukemic cells carrying FLT3 mutation **A.** Schema illustrates the workflow of primary and secondary xenograft AML (patients #4 and #5) in NSG mice. Engrafted AML cells (human CD45 positive cells) from spleen or BM were sorted by flow cytometry (anti-human CD45+). **B.** Bar graph on the left displays the percentage of colony formation of sorted xenograft AML leukemic cells treated with the indicated concentrations of MLN0128. Inhibition significance at each concentration was calculated by Student's t-test as shown on the bar graph on the right.

MLN0128 induced apoptosis in AML progenitor CD34+ cells carrying FLT3-ITD, promoting us to further investigate its effects on the function of FLT3-mutated leukemic stem cells. We performed a colony-forming assay on both primary and secondary xenografted cells derived from two AML samples carrying the FLT3-ITD mutation (Figure 4A; Supplementary Table S1 CyTOF section AML samples #4 and #5). These AML-derived xenograft leukemic cells have been reported to resemble leukemic stem cells, possessing the properties of self-renewal and disease initiation [35]. MLN0128 effectively inhibited colony formation in leukemic cells isolated from bone marrow (BM) and spleen of primary and secondary xenograft AML in a dose-dependent fashion (Figure 4B). At 30 nM, MLN0128 reduced colony formation from 100 ± 3.4 % to 56.13 ± 7.14% (mean ± SD, p = 0.0001); at 300 nM, it reduced colony formation to 9.14 ± 0.97% (p < 0.0001). At the similar dose range, MLN0128 showed minimal effect on normal hematopoietic cells [18, 23].

**Figure 5:**
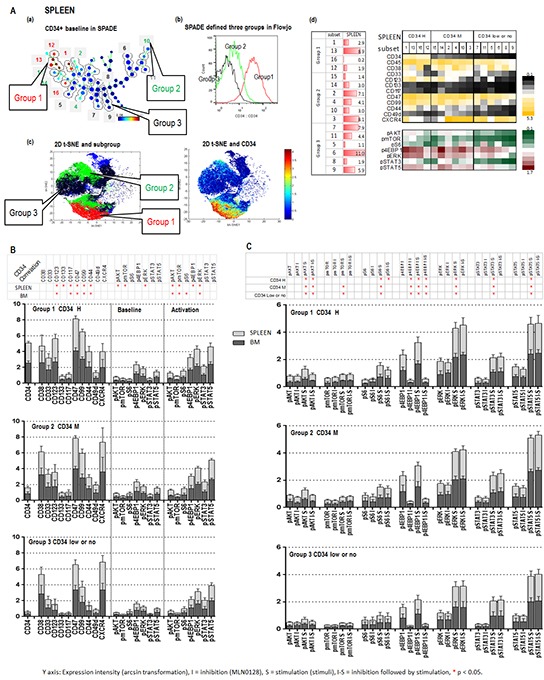
CyTOF identifies the inhibitory effect of MLN0128 on AML-derived xenograft leukemic cells carrying FLT3 mutation **A.** CyTOF analysis of the secondary xenograft AML (#4) recovered from spleen (BM: Supplementary Figure S3). **A (a).** SPADE identified 16 subsets. Subsets with similar intensity of CD34 were grouped as indicated (groups 1, 2, and 3). The defined groups were confirmed by a FlowJo histogram graph **A (b).** and a viSNE 2-D plot **A (c). A (d).** (left): cell frequency of defined subsets; **A (d).** (right): baseline expressions of surface and intracellular molecules in defined subsets of three groups. **B.** The expressions of surface and intracellular molecules in subsets of each group of spleen and BM is displayed in stack bar graphs, and their correlation with CD34 is summarized in a table on top of stack bar graphs. *: significant correlation. **C.** The expression of intracellular molecules before and after stimuli (S = Simulation) and that of MLN0128 (I = inhibition) alone, or followed by stimulation (I-S) in the defined subsets of each group of spleen and BM are displayed in stack bar graphs. Significant stimulation (before and after stimuli), inhibition (before and after MLN0128) and inhibition followed by stimulation (stimuli with or without MLN0128) of intracellular molecules in each group of BM and spleen are summarized in the table on top of stack bar graphs. *: significance (p < 0.05).

We next used CyTOF assay to investigate the inhibitory mechanisms of MLN0128 in AML-derived leukemic cells from spleen and BM of secondary xenograft AML (sample #4). Combined analysis with SPADE, viSNE, and FlowJo identified 16 subpopulations (subsets), which were again grouped based on CD34 expression: group 1, CD34 medium to high (4 subsets); group 2, positive to medium (6 subsets); group 3, low or no expression CD34 (6 subsets) (spleen in Figure 5A a-c; BM in Supplementary Figure S3 a-c). Various cell frequencies and heterogeneous expression of surface and intracellular molecules in the defined subsets of BM and spleen are depicted in the table and heatmap (Figure 5A d; Supplementary Figure S3 d). The frequency of CD34+ cells was higher in spleen than in BM. CD34 expression positively correlated with surface molecules CD123, CD133, CD117, CD47, CD99, and CD44 in spleen subsets; this surface phenotype was associated with both, basal expression of intracellular p-AKT, p-mTOR and p-ERK and with cytokine-induced upregulation of p-AKT, p-mTOR, p-4EBP1 and p-ERK. In BM subsets, CD34 likewise positively correlated with CD123, CD117, CD47, CD99 and CD44, this surface phenotype was associated with upregulated p-AKT, p-mTOR, p-S6 and p-ERK, but not with basal levels of intracellular molecules (Figure 5B). Upregulation of p-AKT, p-ERK, p-STAT3 and p-STAT5 by their cognate stimuli (SCF for p-AKT, GM-CSF for p-ERK and p-STAT3, and IL-6 for p-STAT5) was observed in all subsets of the three groups; however, the highest levels of increasing the expression of p-AKT, p-mTOR, p-4EBP1 and p-ERK in spleen and p-AKT, p-mTOR, p-S6, and p-ERK in BM were observed in subsets of groups 1 and 2 with positive to high CD34. These data suggest that although signaling activation in the xenografted leukemic cells was independent of CD34 expression, CD34+ cells elicited the highest AKT/mTOR and MEK/ERK signaling in response to stimuli (Figure 5B and 5C). MLN0128 was highly selective towards AKT/mTOR signaling; it potently inhibited phosphorylation of both the mTORC1 target 4EBP1 and the mTORC2 target AKT with minimal effects on other pathways in all subsets of the three groups from spleen and BM (Figure 5C; Supplementary Figure S4). Together, our data demonstrate the potent on-target effect of MLN0128 on AML-derived leukemic cells carrying FLT3 mutation.

### Parallel signaling pathways in response to mTORC1/C2 inhibition in AML

**Figure 6:**
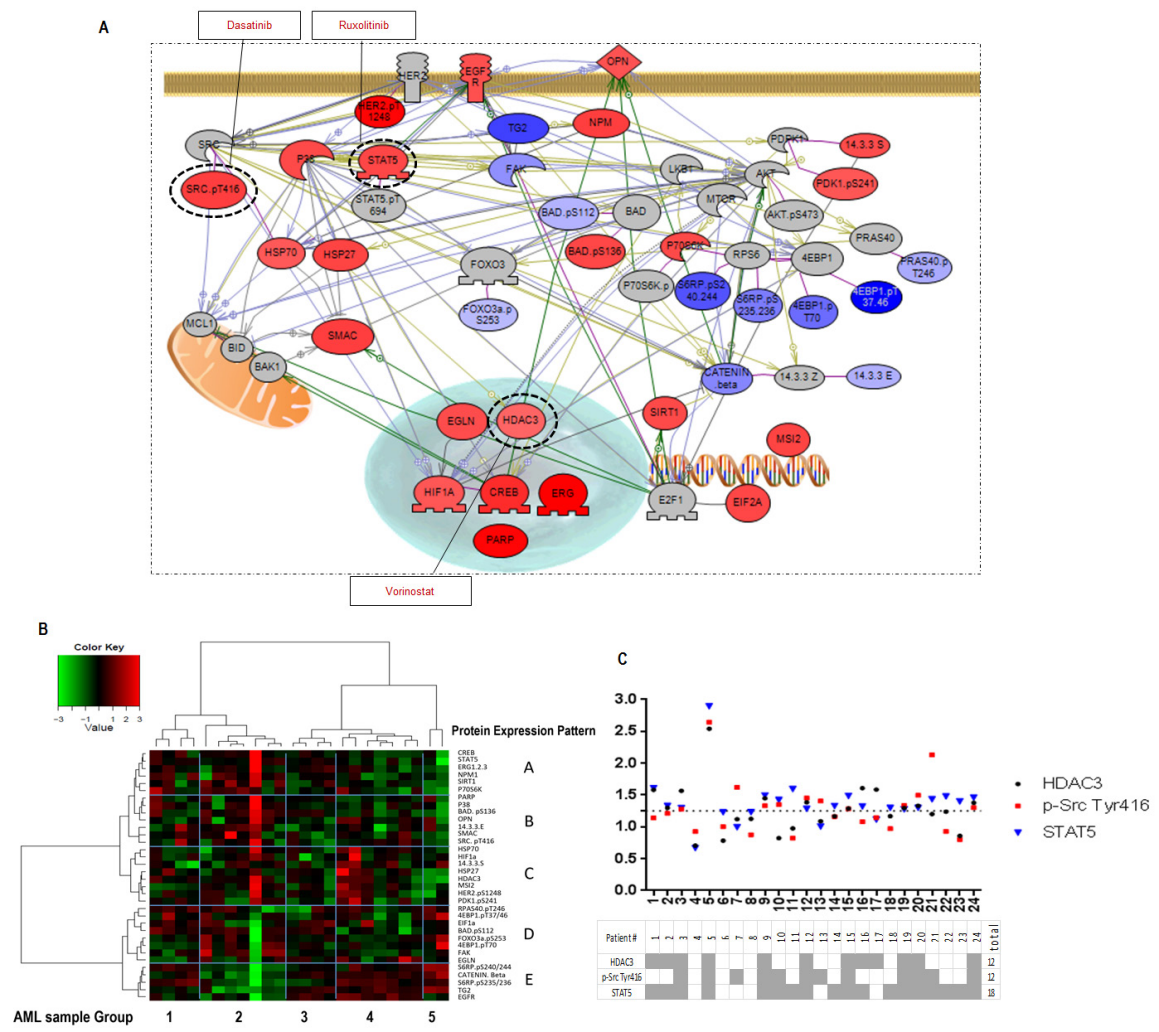
RPPA identifies MLN0128-triggered compensatory signaling pathways **A.** Pathway studio map of the identified 34 proteins significantly affected by MLN0128 in different signaling pathways. Color gradient indicates the fold increase (red) or decrease (blue) in protein intensity after MLN0128 treatment (fold change = protein intensity treated by MLN0128 (log2) - intensity of control (log2)). Proteins that are not significantly modified by MLN0128 but are key molecules in the pathways are colored in gray. Dash-line circles indicate the druggable targets that were identified in the survival pathways triggered by MLN0128 inhibition. The specific inhibitor of each target is shown in a square black box. **B.** Pearson correlation and Ward linkage cluster 34 proteins from 24 primary AML samples; results are summarized in the heatmap. Blue grids highlight the clusters of identified proteins (A-E, right *y*-axis) and the subgroup of samples (1-5, *x*-axis). **C.** MLN0128-triggered upregulation of the druggable targets in each sample were identified at the cutoff ratio ≥ 1.25 (ratio = intensity of a target protein post - pre-exposed to MLN0128). Table summarizes the upregulated targets (gray) in 24 primary AML samples tested.

Our observation that residual AML cells survived after MLN0128 treatment prompted us to investigate alterations in the parallel signaling networks induced by MLN0128 and to identify potential antagonistic mechanisms upon mTOR inhibition. Using RPPA, a high-throughput functional proteomic technology, we measured expression and phosphorylation changes in response to MLN0128 in 151 proteins from 24 primary AML samples with high blast count (Supplementary Table S1 RPPA section; Supplementary Table S2). Statistical analysis identified 34 proteins that were significantly affected by MLN0128 inhibition (Figure 6A; Supplementary Table S4). Consistent with the data found in cell lines, MLN0128 inhibited mTORC1/C2 targets: p-S6 and p-4EBP1 (mTORC1), p-PRAS40 and p-FOXO3a (mTORC2). Unexpectedly, MLN0128 suppressed FAK and TG2, reduced β-catenin expression, and increased SMAC. Simultaneously, MLN0128 inhibition triggered several pro-survival pathways: activation of STAT5, p-SRC, P38 and PDK1 auto-phosphorylation; increased expression of transcription factors CREB and ERG and transcriptional regulators EIF2, HDAC3 and NPM; upregulation of HSP27 and HSP70, PARP, and 14.3.3 sigma; and upregulation of Sirt1 and MSI2. Surprisingly, MLN0128 increased expression of HIF-1α and prolyl hydroxylase EGLN under normoxic conditions, suggesting that compensatory pathways are upregulated upstream of HIF-1α. MLN0128 triggered both phosphorylation (Ser136) and de-phosphorylation (Ser112) of BAD and increased expression of BAD-associated 14.3.3 epsilon. As shown in Figure 6B, additional analysis by Pearson correlation and Ward linkage of these proteins revealed five distinct expression patterns (A-E, *y*-axis) in five sample subgroups (1-5, *x*-axis), indicating heterogeneous responses to MLN0128 among primary AML.

### Therapeutic effect of concurrent blockade of AKT/mTOR signaling and compensatory survival pathways in AML

**Figure 7:**
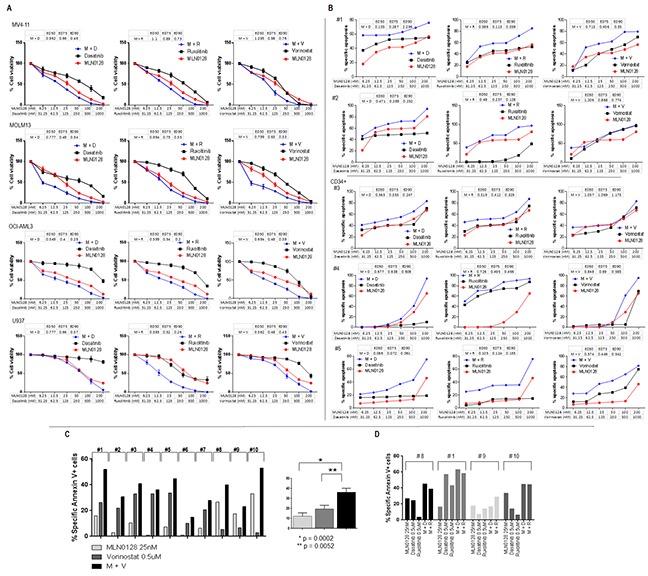
Anti-leukemic efficacy of dual pathway inhibition in AML **A.** AML cell lines were treated with single agent and their combinations at the indicated concentrations for 72 hours. Growth inhibition in cell lines was measured by Cell Titer-Glo® luminescent cell viability assay. Drug efficacy and combination index was calculated using Calcusyn software. **B.** Primary AML samples were treated with single agent and their combinations at the indicated concentrations for 72 hours. Treatment-induced apoptosis in primary samples was determined by flow cytometry of annexin V/DAPI positivity in bulk (#1 and #2) or progenitor cells (CD34+) (#3, #4 and #5). Drug efficacy and combination index was calculated using Calcusyn software. **C** and **D.** Ten primary samples were treated with MLN0128 (25 nM), vorinostat (500 nM) alone or with the combination of vorinostat plus MLN0128 (C); four of the ten samples (# 8, # 1, #9, # 10) were also treated with dasatinib (500 nM), ruxolitinib (500 nM) alone or the combination of MLN0128 plus dasatinib or ruxolitinib (D). Treatment-induced apoptosis at 72 hours was detected by flow cytometry. Specific apoptosis was calculated as described in the Materials and Methods. M: MLN0128; D: dasatinib; R: ruxolitinib; V: vorinostat. Clinical information on primary AML samples shown in 7B and 7C is included in the Supplementary Table S1 combination apoptosis set B and A, respectively.

RPPA analysis identified three druggable resistance targets: HDAC3, p-Src, and STAT5. mTOR blockade by MLN0128 increased the expression of HDAC3 and of p-Src (Tyr416) in 12 samples, and of STAT5 (total) in 18 of 24 samples. Concurrent activation of all three targets was observed in 8 samples (Figure 6C). These targets can be pharmacologically suppressed by vorinostat (HDAC3), dasatinib (p-Src), and ruxolitinib (STAT5). These agents have been tested against leukemia, including AML, both in in vitro and in vivo studies, and in phase I/II trials [36–38]. To examine the pharmacological interactions between targeted agents, we next examined the dose-response of these inhibitors alone and in the combination with MLN0128 in AML cell lines and in primary samples. While anti-leukemia efficacy of these agents used alone is generally lower than that of MLN0128, their combination caused synergistic/additive effects on cell growth inhibition and apoptosis in the majority of cell lines (Figure 7A) and in primary AML samples (Figure 7B), albeit the degree of effect varied depending on a specific combination and samples used (Figure 7A and 7B; Supplementary Figure S6; Supplementary Table S5). Beagle et al. recently reported that the combination of MLN0128 and vorinostat effectively induced apoptosis in primary pediatric B-ALL cells [39]. To further clarify the efficacy of this combination in AML CD34+ cells, we treated 10 additional primary AML samples using a fixed concentration of each agent. The MLN0128 or vorinostat-induced apoptosis in AML CD34+ cells ranged from 1% to 33.3% (mean,12%) or 3% to 33.8% (mean, 19.7%), respectively. Combination treatment elicited a significantly higher apoptosis induction than did single agents (14% to 53%; mean 36.3%) (Figure 7C), with the most effective responses seen in samples #1 (harboring RUNX1 mutation) and #10 (RUNX1, NRAS and EZH2 mutations). To compare the relative efficacy of this combination to other targeted agents, samples #1, #8, #9 and #10 were also treated with the combination of MLN0128 plus dasatinib or ruxolitinib. While increased apoptosis was observed in four samples treated with MLN0128 plus ruxolitinib, and in three samples treated with MLN0128 plus dasatinib (Figure 7D), these were less consistent compared to MLN0128 plus vorinostat.

**Figure 8:**
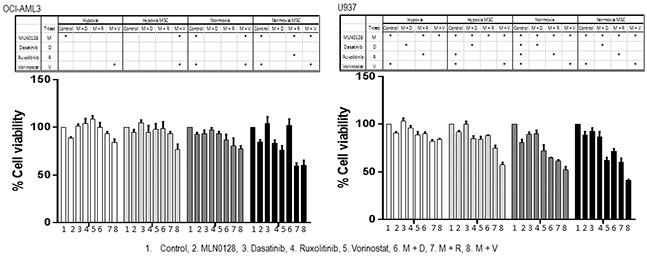
Anti-leukemic efficacy of dual pathway inhibition in AML under conditions mimicking the leukemic microenvironment OCI-AML3 and U937 cells were cultured with or without MSC and treated with MLN0128 (25 nM), dasatinib, ruxolitinib and vorinostat (500 nM) alone and in the combination under hypoxia or normoxia for 48 hours. Growth inhibition of cell lines was measured by Cell Titer-Glo® luminescent cell viability assay. MSC: mesenchymal stromal cells. M: MLN0128; D: dasatinib; R: ruxolitinib; V: vorinostat. *: Combination-treatment significantly inhibited cell growth when compared to single agent used alone. Significance (p ≤ 0.05) was determined by Student's *t*-test.

To further evaluate the combinatorial approaches in AML under the conditions mimicking the hypoxic bone marrow microenvironment, we treated OCI-AML3 and U937 cells and three primary AML samples at the fixed concentration of each agent in co-culture with mesenchymal stromal cells (MSC), under hypoxia (1% oxygen) or normoxia (21% oxygen) (Figure 8; Supplementary Figure S7). The microenvironment altered the drug sensitivity, resulting in the diverse responses of cell lines and primary samples to the single and the combined treatments under different culture conditions. The microenvironment-mediated protection on AML cells from treatment-induced apoptosis was observed in some but not in all samples, and the level of protection on the samples varied depending on the inhibitor. While co-culture with MSC failed to protect cell lines from combination-induced cell death, hypoxia and hypoxia/MSC conditions reduced the efficacy of the treatment. In turn, responses of the 3 primary samples were heterogeneous, with a general trend of reduced cell death under stroma co-cultures and low oxygen conditions (Supplementary Figure S7).

## DISCUSSION

We previously reported that the first-generation asTORi PP242 disrupts mTORC1/C2 in AKT/mTOR signaling, targeting AML cells under conditions that mimic the bone marrow microenvironment [14]. In this study, using MLN0128, a second-generation asTORi, we investigated mTOR inhibition on AML and AML stem/progenitor cells, and studied the inhibition-triggered antagonistic cellular responses. Our findings indicated that MLN0128 is a potent mTORC1/C2 inhibitor that selectively targeted the AKT/mTOR pathway in AML blasts and AML stem/progenitor cells. In turn, mTOR inhibition triggered multiple pro-survival signaling, and three druggable targets were identified to combat these mechanisms. We showed that combined inhibition of these targets and AKT/mTOR signaling facilitates AML cell killing, which supports the use of multi-targeted approaches in AML therapy.

AML is organized in a hierarchy with a small population of leukemia stem cells that are resistant to chemotherapy and propagate disease [40]. Using CyTOF combined with SPADE and viSNE analyses, we were able to dissect the phenotypic heterogeneity of AML and AML stem/progenitor cells, and measure the altered intracellular molecules triggered by stimuli and inhibitors. Thus, this approach is suitable for studying the biology of and molecular effects on rare and heterogeneous cancer stem cells that cannot be easily defined and characterized by conventional methods. Our finding of a positive correlation between expression of CD34 and intracellular signaling of AKT/mTOR and MEK/ERK supports the hypothesis that both anti-apoptosis pathways are critical for AML progenitor cell survival. In addition, our findings that surface clusters, composed of various stem cell molecules are tightly associated with AKT/mTOR and MEK/ERK pathways suggest that both pathways might be critical for the maintenance and regulation of various AML stem/progenitor subpopulations.

Using CyTOF we next characterized AML-derived xenograft leukemic cells carrying FLT3-mutation. The distinct surface and intracellular phenotype identified on the leukemic cells isolated from spleen and BM supports the hypothesis that the xenograft microenvironment (murine BM vs. spleen) influences leukemogenic polarization and lineage differentiation [35]. Furthermore, the same surface phenotype is associated with different intracellular signaling in the basal and activated states, demonstrating that AML stem/progenitor cells contain decoupled surface and signaling signatures, similar to the patterns in pediatric AML cells recently described by Levine et al. [41]. We identified specific intracellular phenotypes, composed of molecules involved in AKT/mTOR and MEK/ERK signaling, that were expressed in xenografted leukemic cells of spleen and BM in the activated cellular state, suggesting that both pathways might be concomitantly required to develop and maintain leukemic cells in different microenvironments. Furthermore, our finding that mTORC1/C2 inhibition by MLN0128 abrogated clonogenic formation supports that xenograft AML stem/progenitor cells proliferation is mainly dependent on AKT/mTOR signaling. Whether this pathway-dependent function is driven by the cytogenetic abnormality of FLT3-mutation and/or by other factors is currently under investigation. Surface expression of CXCR4 and CD44 in leukemia is regulated by the BM microenvironment, while CD34 expression is highly correlated with CD44 but not with CXCR4 in all subsets of spleen and BM, suggesting that each molecule carries a unique function in the interplay between xenograft leukemic cells and their microenvironment. The high selectivity of MLN0128, evidenced by its high potency against mTORC1/2 downstream targets S6, 4EBP1, and AKT and coupled with negligible effects on other signaling pathways, provides the impetus for testing MLN0128 in AML clinical trials, with emphasis on AML patients harboring FLT3 mutations. More importantly, our results raise the possibility that CyTOF technology can be used to monitor the effectiveness of MLN0128 at eliminating AML stem/progenitor cells in the clinical setting.

RPPA data confirmed the inhibitory effect of MLN0128 on mTORC1/C2 substrates; it also revealed that MLN0128 inhibition suppresses FAK, TG2, and β-catenin, all of which are highly expressed in AML and are associated with drug-resistance [42, 43]. FAK was reported to directly interact with AKT/mTOR signaling through its association with and phosphorylation of TSC2 [44] and through phosphorylation by AKT [45]. TSC1 and TSC2 form a complex that negatively regulates mTORC1. MLN0128 prevents the feedback inhibition of TSC1/2 and blocks mTORC2 activation, which might reduce the amount of TSC2 and AKT available to stabilize FAK, resulting in FAK degradation. TG2 is positively associated with FAK [46]; downregulation of FAK by mTORC1/C2 inhibition might affect TG2 expression. Decreased expression of β-catenin might be directly related to MLN0128 inhibition of mTOR and FAK, two upstream mediators of β-catenin. SMAC, a pro-apoptotic molecule in mitochondria, is repressed in AML blast and AML CD34+CD38- cells [47]. mTOR inhibition resulted in increased expression of SMAC, suggesting a negative regulation between AKT/mTOR signaling and functions of SMAC in mitochondria.

Pathway analysis using RPPA data indicated antagonistic responses upon mTORC1/C2 blockade in several intracellular networks, including anti-apoptotic signaling network, DNA repair, mitochondrial maintenance, and stem cell preservation. Five correlation clusters were identified. Each containing key molecules from these compartments suggesting that these responses are network-connected. Furthermore, five sample subgroups each containing the five correlation clusters were identified, with distinct alteration patterns, indicating the presence of sample-specific response signatures. Together, our data demonstrate the cellular network complexity and heterogeneity of responses to mTORC1/C2 inhibition. A similar study in other types of cancer cells treated with the dual PI3K/mTOR inhibitor NVP-BEZ235 demonstrated the expected inhibition of mTORC1 targets of p-4EBP1 and p-S6. However, the survival mechanisms triggered by BEZ235 differed from those of MLN0128 in AML [48], suggesting inhibitor- and cancer cell type-driven specificity.

Our RPPA findings highlight the need to combine mTOR kinase inhibitors with other targeted agents that suppress survival signaling in AML. We identified three druggable targets that were induced upon mTORC1/2 inhibition. We showed that the pan-HDAC inhibitor vorinostat and MLN0128 induced apoptosis in the majority of AML cell lines and in primary AML samples in an additive or synergistic fashion. Several putative mechanisms for the synergistic nature of this combination were recently described in pediatric B-ALL, such as downregulation of FOXO transcription factors and enhanced mitochondrial priming [39]. Whether similar or other mechanisms are involved in AML requires further studies. While vorinostat/MLN0128 combination was most consistently promoting cell death, the toxicity of vorinostat/ panobinostat alone or in the combination with MLN0128 or other mTOR inhibitors has been reported in pre-clinical and clinical studies of T- cell [49] and Hodgkin's lymphoma [50], which warrants in vivo assessment in AML. Similarly, dual inhibition of mTOR with MLN0128 and of Src or JAK/STAT signaling commonly enhanced apoptotic cell death. We observed that combination triggered diverse responses in AML. The minimal effect found in selected cases might result from no or low expression of the target protein specific for an inhibitor, which was not addressed in this study. Recently, Lee and Ye reported that the efficacy of combination treatment is affected by changes in the order and duration of drug exposure [51]. Thus, lack of efficacy in certain combinations may be related to the drug administration sequence or the length of exposure time to single and combined treatment. Altering these conditions might increase target availability, thus improving therapeutic outcomes. Further studies should investigate these possibilities, with the goal of identifying the most effective combinations in individual AML cases. Our preliminary studies conducted under conditions mimicking BM microenvironment emphasize the need for future investigations addressing modulatory effects of stromal cells and oxygen levels on survival of AML blasts.

In summary, our findings indicate that blocking AKT/mTOR signaling with the mTORC1/C2 inhibitor MLN0128 causes growth inhibition and apoptosis in AML cells. High-throughput CyTOF technology revealed that MLN0128 selectively targeted AKT/mTOR signaling in AML stem/progenitor cells. Mapping multiple intracellular signaling pathways in primary AML in response to mTORC1/C2 inhibition identified the compensatory activation of actionable pro-survival pathways. Combinatorial multi-targeted approaches demonstrated various responses in AML samples, suggesting that personalized treatment approaches are warranted. Finally, techniques such as CyTOF or RPPA may assist in profiling the intra-cellular and inter-patient variations in responses to targeted agents, which likely stem from multiple genetic and epigenetic events within individual tumors, and serve as tools for the selection of individualized targeted therapies in AML and other malignancies.

## MATERIALS AND METHODS

### Materials, cell lines and culture, and patient samples

Information about materials, reagents, cell lines and patient samples used in this study is provided in the Supplementary Materials and Methods section.

### Cell viability and apoptosis

Cell viability was measured by Vi-Cell XR cell viability analyzer (Beckman Coulter, Brea, CA) or Cell Titer-Glo® Luminescent cell viability assay (Promega, Madison, WI) as indicated in the text. Apoptosis of AML cell lines and progenitor cells (CD34, BD Pharmingen) was analyzed by flow cytometry of annexin V (Roche Diagnostics, Indianapolis, IN) and propidium iodide (PI) or DAPI positivity (PI and DAPI, Sigma). The extent of drug-specific apoptosis was assessed by the formula: % specific apoptosis = (test − control)/(100 − control) × 100. Where “test” is the percentage of annexin V/PI or DAPI positivity in treated cells, and “control” is the percentage of annexin V/PI or DAPI positivity in untreated cells (spontaneous apoptosis) [52]. For the dose response of single and combination drug studies, drug efficacy and combination index were calculated by Calcusyn software (Biosoft, Cambridge, UK).

### Western blot analysis

Protein expression in drug-treated cells was determined by Western blot as described previously [14]. Protein signals in Western blot were detected by the Odyssey Infrared Imaging System (LI-COR Biosciences, Lincoln, NE) and quantified using Odyssey software (version 3.0, LI-COR Biosciences). Antibodies used in the Western blot are listed in the Supplementary Materials and Methods.

### Reverse-phase protein array (RPPA)

Peripheral blood samples were collected from 24 AML patients with high blast count (Supplementary Table S1 RPPA section). Mononuclear cells were separated by Ficoll gradient and exposed to 0.1 mM MLN0128 for 6 hours in vitro. Treated cells were lysed and subjected to RPPA using previously described and validated methods [53]. The Antibodies used to detect 151 proteins in RPPA are listed in Supplementary Table S2. Tukey's test was used to determine the differential protein expression or phosphorylation level of each protein that was associated with mTOR inhibition. Significantly altered proteins were mapped into the signaling network using Pathway Studio v9.0 (Elsevier, NY) and displayed in a heatmap generated by Pearson correlation and the Ward linkage method.

### Primary AML transplantation in NOD/SCID mice

Serial transplantation of primary AML in NSG mice (Jackson Laboratory, Bar Harbor, Maine) was performed as described previously [54]. Briefly, primary AML cells or primary xenograft AML cells were intravenously injected into irradiated (300 cGy) NOD/SCID mice of age 6-8 weeks at a concentration of 0.5 × 10^6^ cells/mouse. Engraftment was confirmed by flow cytometry to check mouse circulating human CD45 positive cells (anti-human CD45-PE, BD Bioscience). Mice were sacrificed when 80 % of engraftment was achieved. Engrafted cells were isolated from mouse BM and spleen, then sorted on human CD45+/DAPI-. Sorted cells were used for colony formation and CyTOF assay.

### Clonogenic assay

The colony-forming assay was performed as described previously [55]. Sorted cells from mice with primary and secondary xenografted AML were plated in methylcellulose supplemented with various human recombinant growth factors (MethoCult™ H4435 Enriched StemCell Technology, Vancouver, BC, Canada). MLN0128 at 30 nM, 100 nM, or 300 nM or DMSO alone was added at the beginning of the cultures. Triplicate cultures of each concentration were grown in 35-mm Petri dishes and incubated at 37°C in a humidified atmosphere with 5% CO_2_. AML blast colonies were assessed by light microscopy between days 8 and 10.

### CyTOF assay and analyses

The CyTOF assay was performed as described in a previous report [56] and in the Supplementary Materials and Methods. Data collected from CyTOF were normalized using a MATLAB-based software called Bead-normalization [57]. Normalized data were processed by FlowJo v10 by gating on Ir-positive cells to eliminate interference from beads and background noise caused by reagents or instrument. Processed data were then analyzed by SPADE, to build a tree structure by computing K-means clustering algorithm and minimal spanning tree algorithm on surface markers. Subpopulations/subsets identified by SPADE were cross-validated by viSNE and FlowJo v10 and then further analyzed by GraphPad Prism v6. Pearson and Spearman correlations were used to calculate expression correlations of the indicated molecules. Data were considered significant when results of each correlation were in agreement (R ≥ 0.5 and p < = 0.05). One – way ANOVA followed by Tukey post-hoc test were used to calculate significant stimulation (SCF or stimuli indicated in the text and figures) or inhibition (MLN0128) on SPADE defined cell subsets.

### Statistical analyses

Student's *t*-test and one – way ANOVA followed by Tukey's post-hoc test were used to calculate p values for experiments with single-paired or multiple-paired comparisons. Statistical analyses associated with particular methods are indicated in the text. A p value ≤ 0.05 was considered statistically significant. All cell lines experiments were performed in triplicate unless stated otherwise.

### Summary sentence

Targeting of mTORC1/C2 by the active-site mTOR kinase inhibitor MLN0128 leads to anti-leukemic effects in AML and AML stem/progenitor cells.

## SUPPLEMENTARY MATERIALS AND METHODS







## References

[1] Sabatini DM (2012). Control of growth by the mTOR pathway. Faseb J.

[2] Laplante M, Sabatini DM (2012). mTOR signaling. Csh Perspect Biol.

[3] Estey EH, Dohner H (2006). Acute myeloid leukaemia. Lancet.

[4] Dohner H, Estey EH, Amadori S, Appelbaum FR, Buchner T, Burnett AK, Dombret H, Fenaux P, Grimwade D, Larson RA, Lo-Coco F, Naoe T, Niederwieser D (2010). Diagnosis and management of acute myeloid leukemia in adults: recommendations from an international expert panel, on behalf of the European LeukemiaNet. Blood.

[5] Altman JK, Platanias LC (2008). Exploiting the mammalian target of rapamycin pathway in hematologic malignancies. Curr Opin Hematol.

[6] Altman JK, Sassano A, Platanias LC (2011). Targeting mTOR for the treatment of AML. New agents and new directions. Oncotarget.

[7] Zoncu R, Efeyan A, Sabatini DM (2011). mTOR: from growth signal integration to cancer, diabetes and ageing. Nature reviews Molecular cell biology.

[8] Hsu PP, Kang SA, Rameseder J, Zhang Y, Ottina KA, Lim D, Peterson TR, Choi YM, Gray NS, Yaffe MB, Marto JA, Sabatini DM (2011). The mTOR-Regulated Phosphoproteome Reveals a Mechanism of mTORC1-Mediated Inhibition of Growth Factor Signaling. Science.

[9] Zeng Z, Sarbassov DD, Samudio IJ, Yee KW, Munsell MF, Jackson E, Giles FJ, Sabatini DM, Andreeff M, Konopleva M (2007). Rapamycin derivatives reduce mTORC2 signaling and inhibit AKT activation in AML. Blood.

[10] Sarbassov DD, Guertin DA, Ali SM, Sabatini DM (2005). Phosphorylation and regulation of Akt/PKB by the rictor-mTOR complex. Science.

[11] Feldman ME, Apsel B, Uotila A, Loewith R, Knight ZA, Ruggero D, Shokat KM (2009). Active-Site Inhibitors of mTOR Target Rapamycin-Resistant Outputs of mTORC1 and mTORC2. Plos Biol.

[12] Thoreen CC, Kang SA, Chang JW, Liu QS, Zhang JM, Gao Y, Reichling LJ, Sim TB, Sabatini DM, Gray NS (2009). An ATP-competitive Mammalian Target of Rapamycin Inhibitor Reveals Rapamycin-resistant Functions of mTORC1. J Biol Chem.

[13] Janes MR, Limon JJ, So LM, Chen J, Lim RJ, Chavez MA, Vu C, Lilly MB, Mallya S, Ong ST, Konopleva M, Martin MB, Ren PD, Liu Y, Rommel C, Fruman DA (2010). Effective and selective targeting of leukemia cells using a TORC1/2 kinase inhibitor. Nat Med.

[14] Zeng ZH, Shi YX, Tsao T, Qiu YH, Kornblau SM, Baggerly KA, Liu WB, Jessen K, Liu Y, Kantarjian H, Rommel C, Fruman DA, Andreeff M, Konopleva M (2012). Targeting of mTORC1/2 by the mTOR kinase inhibitor PP242 induces apoptosis in AML cells under conditions mimicking the bone marrow microenvironment. Blood.

[15] Gokmen-Polar Y, Liu Y, Toroni RA, Sanders KL, Mehta R, Badve S, Rommel C, Sledge GW (2012). Investigational drug MLN0128, a novel TORC1/2 inhibitor, demonstrates potent oral antitumor activity in human breast cancer xenograft models. Breast cancer research and treatment.

[16] Kang MH, Reynolds CP, Maris JM, Gorlick R, Kolb EA, Lock R, Carol H, Keir ST, Wu J, Lyalin D, Kurmasheva RT, Houghton PJ, Smith MA (2014). Initial testing (stage 1) of the investigational mTOR kinase inhibitor MLN0128 by the pediatric preclinical testing program. Pediatric blood & cancer.

[17] Ingels A, Zhao H, Thong AE, Saar M, Valta MP, Nolley R, Santos J, Peehl DM (2014). Preclinical trial of a new dual mTOR inhibitor, MLN0128, using renal cell carcinoma tumorgrafts. International journal of cancer.

[18] Janes MR, Vu C, Mallya S, Shieh MP, Limon JJ, Li LS, Jessen KA, Martin MB, Ren P, Lilly MB, Sender LS, Liu Y, Rommel C, Fruman DA (2013). Efficacy of the investigational mTOR kinase inhibitor MLN0128/INK128 in models of B-cell acute lymphoblastic leukemia. Leukemia.

[19] Slotkin EK, Patwardhan PP, Vasudeva SD, Stanchina E, Tap WD, Schwartz GK (2015). MLN0128, an ATP-competitive mTOR kinase inhibitor with potent in vitro and in vivo antitumor activity, as potential therapy for bone and soft-tissue sarcoma. Molecular cancer therapeutics.

[20] Janes MR, Fruman DA (2010). Targeting TOR dependence in cancer. Oncotarget.

[21] Wander SA, Hennessy BT, Slingerland JM (2011). Next-generation mTOR inhibitors in clinical oncology: how pathway complexity informs therapeutic strategy. J Clin Invest.

[22] Paplomata E, O'Regan R (2014). The PI3K/AKT/mTOR pathway in breast cancer: targets, trials and biomarkers. Therapeutic advances in medical oncology.

[23] Rahmani M, Aust MM, Hawkins E, Parker RE, Ross M, Kmieciak M, Reshko LB, Rizzo KA, Dumur CI, Ferreira-Gonzalez A, Grant S (2015). Co-administration of the mTORC1/TORC2 inhibitor INK128 and the Bcl-2/Bcl-xL antagonist ABT-737 kills human myeloid leukemia cells through Mcl-1 down-regulation and AKT inactivation. Haematologica.

[24] Hope KJ, Jin L, Dick JE (2004). Acute myeloid leukemia originates from a hierarchy of leukemic stem cell classes that differ in self-renewal capacity. Nature immunology.

[25] Kurmasheva RT, Huang S, Houghton PJ (2006). Predicted mechanisms of resistance to mTOR inhibitors. British journal of cancer.

[26] Sparks CA, Guertin DA (2010). Targeting mTOR: prospects for mTOR complex 2 inhibitors in cancer therapy. Oncogene.

[27] Janes MR, Rommel C (2011). Next-generation flow cytometry. Nat Biotechnol.

[28] Tibes R, Qiu Y, Lu Y, Hennessy B, Andreeff M, Mills GB, Kornblau SM (2006). Reverse phase protein array: validation of a novel proteomic technology and utility for analysis of primary leukemia specimens and hematopoietic stem cells. Molecular cancer therapeutics.

[29] Qiu P, Simonds EF, Bendall SC, Gibbs KD, Bruggner RV, Linderman MD, Sachs K, Nolan GP, Plevritis SK (2011). Extracting a cellular hierarchy from high-dimensional cytometry data with SPADE. Nat Biotechnol.

[30] Amir ED, Davis KL, Tadmor MD, Simonds EF, Levine JH, Bendall SC, Shenfeld DK, Krishnaswamy S, Nolan GP, Pe'er D (2013). viSNE enables visualization of high dimensional single-cell data and reveals phenotypic heterogeneity of leukemia. Nat Biotechnol.

[31] Du X, Ho M, Pastan I (2007). New immunotoxins targeting CD123, a stem cell antigen on acute myeloid leukemia cells. Journal of immunotherapy.

[32] Vercauteren SM, Sutherland HJ (2001). CD133 (AC133) expression on AML cells and progenitors. Cytotherapy.

[33] Jaiswal S, Jamieson CH, Pang WW, Park CY, Chao MP, Majeti R, Traver D, van Rooijen N, Weissman IL (2009). CD47 is upregulated on circulating hematopoietic stem cells and leukemia cells to avoid phagocytosis. Cell.

[34] Zhang PJ, Barcos M, Stewart CC, Block AW, Sait S, Brooks JJ (2000). Immunoreactivity of MIC2 (CD99) in acute myelogenous leukemia and related diseases. Modern pathology.

[35] Risueno RM, Campbell CJ, Dingwall S, Levadoux-Martin M, Leber B, Xenocostas A, Bhatia M (2011). Identification of T-lymphocytic leukemia-initiating stem cells residing in a small subset of patients with acute myeloid leukemic disease. Blood.

[36] Garcia-Manero G, Yang H, Bueso-Ramos C, Ferrajoli A, Cortes J, Wierda WG, Faderl S, Koller C, Morris G, Rosner G, Loboda A, Fantin VR, Randolph SS (2008). Phase 1 study of the histone deacetylase inhibitor vorinostat (suberoylanilide hydroxamic acid [SAHA]) in patients with advanced leukemias and myelodysplastic syndromes. Blood.

[37] Verstovsek S, Tefferi A, Cortes J, O'Brien S, Garcia-Manero G, Pardanani A, Akin C, Faderl S, Manshouri T, Thomas D, Kantarjian H (2008). Phase II study of dasatinib in Philadelphia chromosome-negative acute and chronic myeloid diseases, including systemic mastocytosis. Clin Cancer Res.

[38] Naqvi K, Verstovsek S, Kantarjian H, Ravandi F (2011). A potential role of ruxolitinib in leukemia. Expert Opin Investig Drugs.

[39] Beagle BR, Nguyen DM, Mallya S, Tang SS, Lu M, Zeng Z, Konopleva M, Vo TT, Fruman DA (2015). mTOR kinase inhibitors synergize with histone deacetylase inhibitors to kill B-cell acute lymphoblastic leukemia cells. Oncotarget.

[40] Ramdass B, Chowdhary A, Koka PS (2013). Hematological malignancies: disease pathophysiology of leukemic stem cells. Journal of stem cells.

[41] Levine JH, Simonds EF, Bendall SC, Davis KL, Amir el AD, Tadmor MD, Litvin O, Fienberg HG, Jager A, Zunder ER, Finck R, Gedman AL, Radtke I, Downing JR, Pe'er D, Nolan GP (2015). Data-Driven Phenotypic Dissection of AML Reveals Progenitor-like Cells that Correlate with Prognosis. Cell.

[42] Pierce A, Whetton AD, Meyer S, Ravandi-Kashani F, Borthakur G, Coombes KR, Zhang NX, Kornblau S (2013). Transglutaminase 2 expression in acute myeloid leukemia: Association with adhesion molecule expression and leukemic blast motility. Proteomics.

[43] Despeaux M, Chicanne G, Rouer E, De Toni-Costes F, Bertrand J, Mansat-De Mas R, Vergnolle N, Eaves C, Payrastre B, Girault JA, Racaud-Sultan C (2012). Focal Adhesion Kinase Splice Variants Maintain Primitive Acute Myeloid Leukemia Cells Through Altered Wnt Signaling. Stem Cells.

[44] Gan B, Yoo Y, Guan JL (2006). Association of focal adhesion kinase with tuberous sclerosis complex 2 in the regulation of s6 kinase activation and cell growth. J Biol Chem.

[45] Wang S, Basson MD (2011). Protein kinase B/AKT and focal adhesion kinase: two close signaling partners in cancer. Anti-cancer agents in medicinal chemistry.

[46] Verma A, Mehta K (2007). Tissue transglutaminase-mediated chemoresistance in cancer cells. Drug resistance updates.

[47] Kornblau SM, Qutub A, Yao H, York H, Qiu YH, Graber D, Ravandi F, Cortes J, Andreeff M, Zhang NX, Coombes KR (2013). Proteomic Profiling Identifies Distinct Protein Patterns in Acute Myelogenous Leukemia CD34+CD38-Stem-Like Cells. Plos One.

[48] Muranen T, Selfors LM, Worster DT, Iwanicki MP, Song L, Morales FC, Gao S, Mills GB, Brugge JS (2012). Inhibition of PI3K/mTOR leads to adaptive resistance in matrix-attached cancer cells. Cancer cell.

[49] Oki Y, Buglio D, Fanale M, Fayad L, Copeland A, Romaguera J, Kwak LW, Pro B, de Castro Faria S, Neelapu S, Fowler N, Hagemeister F, Zhang J, Zhou S, Feng L, Younes A (2013). Phase I study of panobinostat plus everolimus in patients with relapsed or refractory lymphoma. Clin Cancer Res.

[50] Younes A, Sureda A, Ben-Yehuda D, Zinzani PL, Ong TC, Prince HM, Harrison SJ, Kirschbaum M, Johnston P, Gallagher J, Le Corre C, Shen A, Engert A (2012). Panobinostat in patients with relapsed/refractory Hodgkin's lymphoma after autologous stem-cell transplantation: results of a phase II study. J Clin Oncol.

[51] Lee MJ, Ye AS, Gardino AK, Heijink AM, Sorger PK, MacBeath G, Yaffe MB (2012). Sequential application of anticancer drugs enhances cell death by rewiring apoptotic signaling networks. Cell.

[52] Kojima K, Konopleva M, McQueen T, O'Brien S, Plunkett W, Andreeff M (2006). Mdm2 inhibitor Nutlin-3a induces p53-mediated apoptosis by transcription-dependent and transcription-independent mechanisms and may overcome Atm-mediated resistance to fludarabine in chronic lymphocytic leukemia. Blood.

[53] Kornblau SM, Tibes R, Qiu YH, Chen W, Kantarjian H, Andreeff M, Coombes KR, Mills GB (2009). Functional proteomic profiling of AML predicts response and survival. Blood.

[54] Bonnet D, Dick JE (1997). Human acute myeloid leukemia is organized as a hierarchy that originates from a primitive hematopoietic cell. Nat Med.

[55] Zeng Z, Samudio IJ, Zhang W, Estrov Z, Pelicano H, Harris D, Frolova O, Hail N, Chen W, Kornblau SM, Huang P, Lu Y, Mills GB, Andreeff M, Konopleva M (2006). Simultaneous inhibition of PDK1/AKT and Fms-like tyrosine kinase 3 signaling by a small-molecule KP372-1 induces mitochondrial dysfunction and apoptosis in acute myelogenous leukemia. Cancer research.

[56] Han L, Qiu P, Zeng Z, Jorgensen JL, Mak DH, Burks JK, Schober W, McQueen TJ, Cortes J, Tanner SD, Roboz GJ, Kantarjian HM, Kornblau SM, Guzman ML, Andreeff M, Konopleva M (2015). Single-cell mass cytometry reveals intracellular survival/proliferative signaling in FLT3-ITD-mutated AML stem/progenitor cells. Cytometry Part A.

[57] Finck R, Simonds EF, Jager A, Krishnaswamy S, Sachs K, Fantl W, Pe'er D, Nolan GP, Bendall SC (2013). Normalization of mass cytometry data with bead standards. Cytometry Part A.

