# Supplementary material for: MLN0128, a novel mTOR kinase inhibitor, disrupts survival signaling and triggers apoptosis in AML and AML stem/ progenitor cells
Source: Oncotarget. 2016 Jul 4;7(34):55083–97. doi: 10.18632/oncotarget.10397 (PMC5342403; doi:10.18632/oncotarget.10397)
Supplement: Supplementary file 1 [file oncotarget-07-55083-s001.pdf]

MLN0128, a novel mTOR kinase inhibitor, disrupts survival signaling and triggers apoptosis in AML and AML stem/progenitor cells

SUPPLEMENTARY MATERIALS AND METHODS

Materials

MLN0128 was provided by Millennium Pharmaceuticals (Cambridge, MA), vorinostat and dasatinib were purchased from LC Laboratories (Woburn, MA), ruxolitinib (INCB018424) was from Selleck (Boston, MA), and rapamycin was purchased from Sigma-Aldrich (St. Louis, MO). Antibodies used in Western blot and flow cytometry analyses were obtained from Cell Signaling Technology (Danvers, MA), Abcam (Cambridge, MA), BD Pharmingen (Franklin Lakes, NJ), eBioscience (San Diego, CA), and Biolegend (San Diego, CA). All metal-conjugated antibodies, metal-labeling kits, and Ir-intercalator used in the CyTOF experiments were purchased from Fluidigm Corporation (South San Francisco, CA). Paraformaldehyde (PFA) was from Electron Microscopy Sciences (Hatfield, PA)**.** Stem cell growth factor (SCF) and IL-6 were purchased from Thermo Fisher Scientific (Grand Island, NY); GM-CSF was from Immunex Corporation (Seattle, WA).

Cell lines and culture

OCI-AML3 was provided by Dr. Mark Minden (Ontario Cancer Institute, Toronto, Ontario, Canada). U937, MV4-11, and MOLM13 were purchased from American Type Culture Collection (Manassas, VA). All cell lines were cultured in RPMI 1640 medium (Mediatech, Inc. Manassas, VA) supplemented with 10% fetal bovine serum (FBS; Gemini Bio-Products, Woodland, CA), 1 mmol/L L-glutamine and 50 μg/mL penicillin-streptomycin (all from Thermo Fisher Scientific) and incubated at 37°C in 21% O_2 and 5% CO_2 condition. For experiments involving hypoxic conditions (1% O_2), cells were incubated in the hypoxic Workstation INVIVO2 400 (Ruskinn Technology, Bridgend, Mid Glamorgan CF31 3TB, United Kingdom) for the indicated time period.

Human subjects

Peripheral blood and bone marrow samples were obtained from AML patients after informed consent had been obtained in accordance with The University of Texas MD Anderson Cancer Center (Houston, Texas) institutional review board regulations (Supplementary Table S1). Mononuclear cells in primary samples were separated using a Ficoll-Hypaque density gradient centrifugation (Sigma-Aldrich). Isolated cells were cultured in RPMI 1640 medium containing 10% FBS, 1 mmol/L L-glutamine and 50 μg/mL penicillin/streptomycin. Normal human adult bone marrow mesenchymal stromal cells (MSC) were isolated from bone marrow aspirates from the iliac crest of healthy volunteers after informed consent according to institutional guidelines of the Declaration of Helsinki. MSC isolation, expansion and purification were performed according to a previously described protocol (1); enriched MSC were maintained in α-minimum essential medium (α-MEM; Thermo Fisher Scientific) supplemented with 20% FBS, 1 mmol/L L-glutamine and 50 μg/mL penicillin/streptomycin.

Co-culture of leukemic cells and MSC

MSCs were plated and cultured in α-MEM medium (20% FBS, 1 mmol/L L-glutamine and 50 μg/mL penicillin/streptomycin) overnight. After removal of the culture medium, leukemic cells were seeded on top of MSC at a ratio of 1:100 (MSC: leukemic cells) and co-cultured in RPMI-1640 medium (10% FBS, 1 mmol/L L-glutamine and 50 μg/mL penicillin/streptomycin) for 3 hours, after which co-cultures were exposed to single agents or their combinations at the indicated concentrations in normoxia or hypoxia for 48 hours.

To determine the treatment effect on the co-cultured leukemic cells in normoixa or hypoxia conditions, AML cells were harvested and separated from MSC according to a previously described protocol (14). Cell viability was measured by Cell Titer-Glo® Luminescent viability assay. Apoptosis induction on AML cells (CD45+/CD34+) was analyzed by flow cytometry (CD45, BD Pharmingen). Specific apoptosis was calculated as described in the main text.

Western blot analysis

Antibodies against human p-AKT (Ser473) (phosphorylated AKT(Ser473)), p-AKT(Thr308), p-GSKα/β(Ser21/9), p-S6K(Ser240/244), p-PRAS40(Ser246), p-4EBPGSK b1(Thr37/46), AKT, S6K, 4EBP1, PRAS40, and GSKβ were purchased from Cell Signaling Technology. GAPDH, used as a loading control, was purchased from Abcam.

CyTOF assay

Cryopreserved peripheral blood mononuclear cells were thawed and rested in 20% FBS RPMI medium that contained heparin (0.1 mg/ml, Alfa Aesar), $MgSO_4$ (100 mM) and DNase (10 units/ml) (both from Thermo Fisher Scientific) for 1 hour before use. Freshly sorted cells from mouse bone marrow or spleen were rested in 20% FBS RPMI medium for 2 hours before use. Cells with 95% or higher viability were treated with 0.1 µM MLN0128 for 60 minutes, stimulated with stem cell factor (SCF; 100 ng/mL), or GM-CSF (100 U/mL), or IL-6 (100 ng/mL) for 5 minutes, and fixed with paraformaldehyde at a final concentration of 1.6% at room temperature for 10 minutes. Cells were washed three times with washing buffer (phosphate-buffered saline [PBS], 0.5% bovine serum albumin [BSA], and 0.02% sodium azide) and resuspended in staining buffer (0.5% BSA-PBS) at a concentration of 1 million cells/35 µl. Antibody mix (15 µl) against cell surface molecules (Supplementary Table S3) was added to the resuspended cells to the final staining volume of 50 µL. Cells were incubated for 30 minutes at room temperature and washed twice with washing buffer. The pellet was then resuspended in 300 µl of 1.6% PFA-PBS, incubated for 10 minutes at room temperature, and spun down at 1000 g for 3 minutes at 4°C. Cells were permeabilized by 1 ml of cold 70% methanol for 10 minutes on ice, washed three times with washing buffer, and resuspended in staining buffer (1 million cells/35 µl) before 15 µl of antibody mix against intracellular molecules (Supplementary Table S3) was added. Cells were stained for 30 minutes at room temperature. Stained cells were washed three times with washing buffer and incubated with 0.5 ml of 1.6% PFA-PBS containing 250 nM Ir-intercalator for 20 minutes at room temperature or overnight at 4°C. Cells were then washed two times with washing buffer and filtered with distilled water through blue-capped cell strainer tubes (Thermo Fisher Scientific) to remove clumps. Filtered cells were counted and spun down, leaving cell pellets (1×10^6) in a residual volume of distilled water (50 µl). Eu151/153 beads were added to cells before the cells were transferred to a 96-deep well plate. An autosampler device (Fluidigm Corporation) was used to apply the sample to the CyTOF instrument.

REFERENCES

1. Kojima K, McQueen T, Chen Y, et al. p53 activation of mesenchymal stromal cells partially abrogates microenvironment-mediated resistance to FLT3 inhibition in AML through HIF-1alpha-mediated down-regulation of CXCL12. Blood 2011;118:4431-9.

SUPPLEMENTARY FIGURES AND TABLES

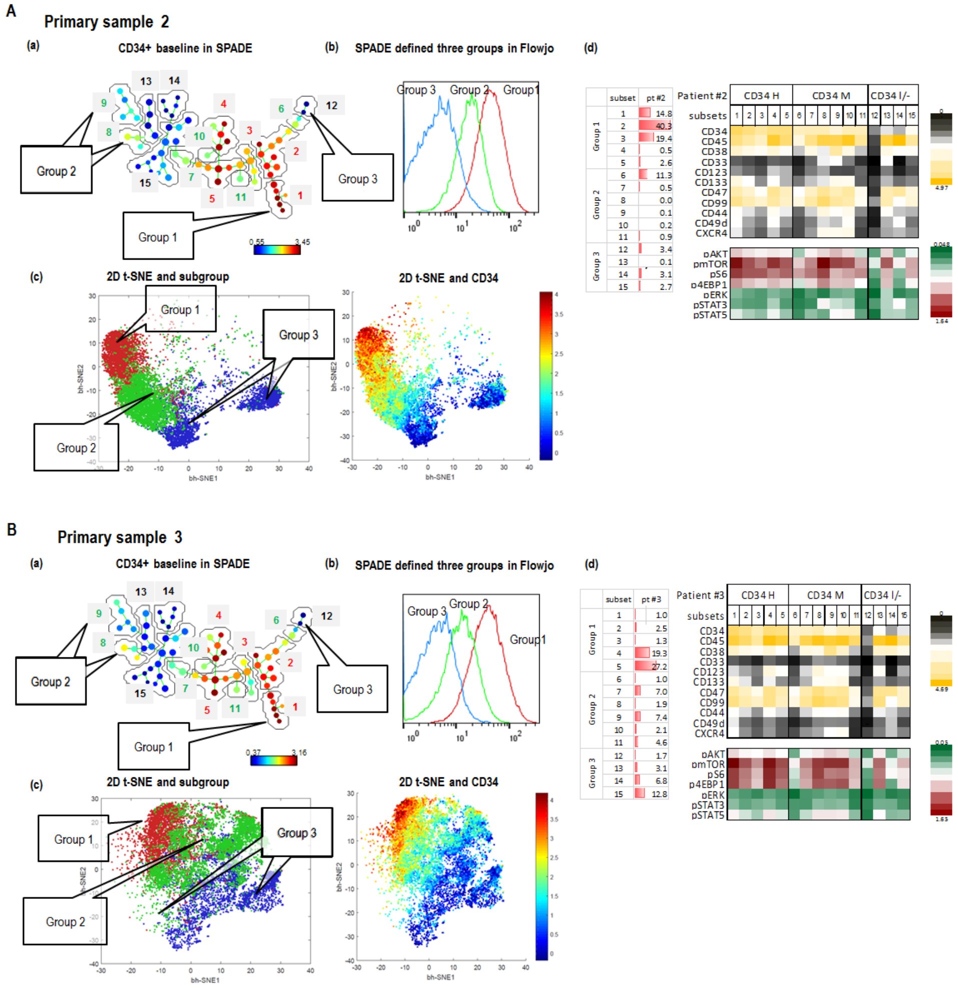

Supplementary Figure S1: CyTOF identifies the effect of MLN0128 on primary AML stem/progenitor cells (#2 and #3), related to Figure 3. CyTOF, SPADE and viSNE analysis of the primary AML sample #2 **A.** and #3. **B.** (a) SPADE displays primary sample CD34 baseline expression. Subsets with similar intensity of CD34 were grouped as indicated (groups 1, 2, and 3) with high, medium and low or no expression of CD34. The defined groups were confirmed by FlowJo in histogram graph (b) and by viSNE in 2-D plot (c) showing each group location, geometry (left), and CD34 intensity and distribution (right). (d, left): cell frequency of the defined subsets; (d, right): the baseline expressions of surface and intracellular molecules of the defined subsets.

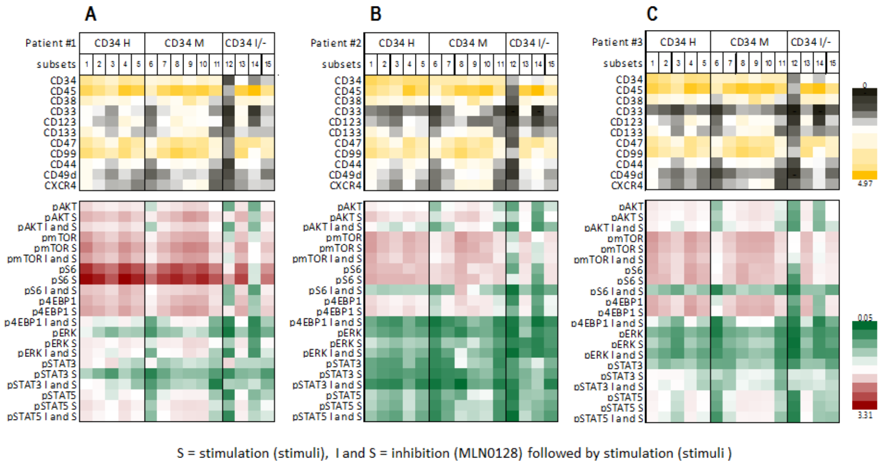

Supplementary Figure S2: Heatmap summarizes the expression intensity of surface and intracellular molecules in subsets of each group (groups 1, 2, and 3) of three primary samples (1, 2 and 3). A, B and **C.** Heatmap of surface markers (black to yellow) was scaled based on the baseline expression intensities of all three samples. Heatmap of intracellular molecules (green to red) was scaled on the expression intensities of baseline, stimulation (stimuli, "S") and inhibition (MLN0128, "I") of intracellular markers of all three samples.

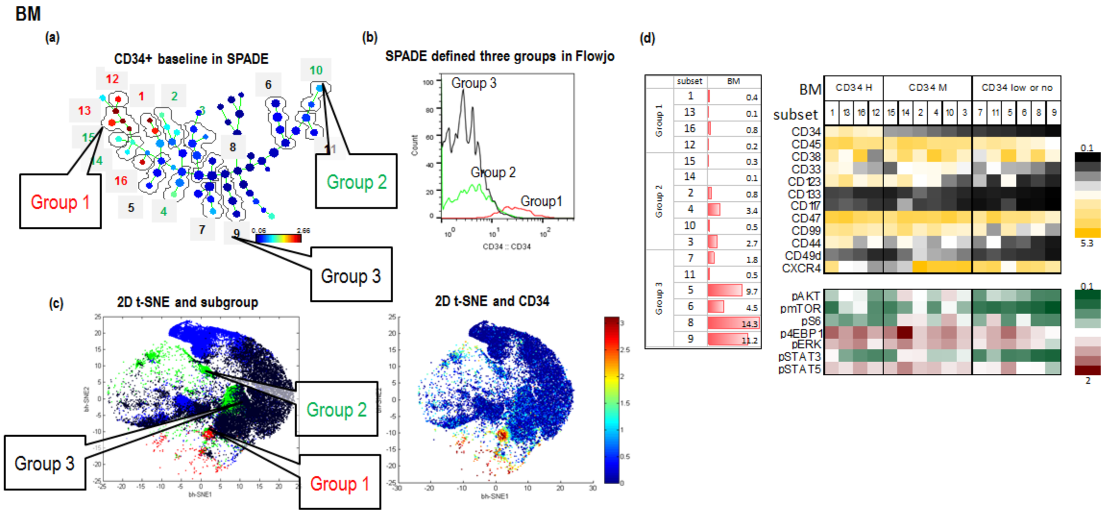

Supplementary Figure S3: CyTOF identifies the effect of MLN0128 on AML-derived xenograft leukemic cells, related to Figure 5. SPADE and viSNE analysis of the secondary xenograft primary AML cells (#4) recovered from BM. **a.** SPADE displays CD34 baseline expression. 16 subsets were identified; subsets with similar intensity of CD34 were grouped (groups 1, 2, and 3) as indicated with high, medium and low or no expression of CD34. The defined groups were confirmed by FlowJo in a histogram graph **b.** and by viSNE in a 2-D plot **c.** showing each group location, geometry (left), CD34 intensity and distribution (right). **d. (left):** cell frequency of defined subsets; **d. (right):** baseline expression of surface and intracellular molecules in subsets of each defined group.

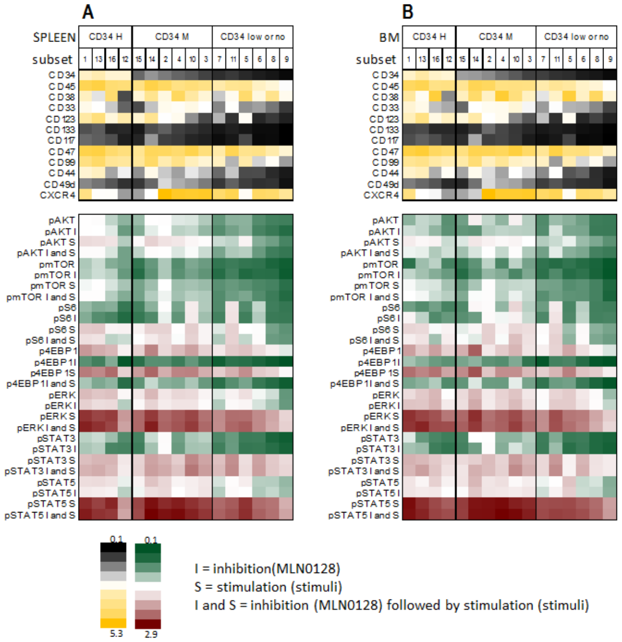

Supplementary Figure S4: Heatmap summarizes the expression intensity of surface and intracellular molecules in subsets of each group (groups 1, 2, and 3) of samples recovered from spleen A. and BM **B.** Heatmap of surface markers (black to yellow) was scaled on the baseline expression intensities of both spleen and bone marrow samples. Heatmap of intracellular molecules (green to red) was scaled on the expression intensities of baseline, inhibition (MLN0128, “I”), stimulation (stimuli, “S”) and inhibition followed by simulation (“I and S”) of both spleen and bone marrow samples.

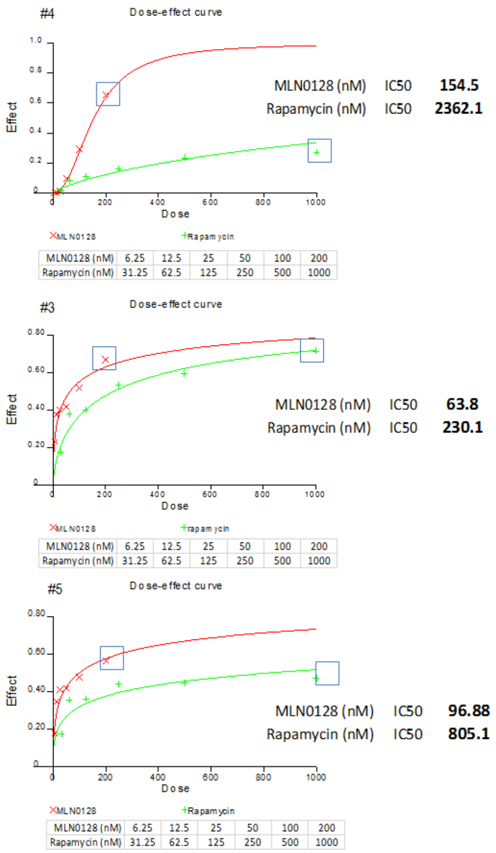

Supplementary Figure S5: Anti-leukemic efficacy of MLN0128 and rapamycin in primary AML. Primary AML samples were treated with MLN0128 or rapamycin at the indicated concentration for 72 hours. Apoptosis induction in bulk (#4) and progenitor CD34+ cells (#3 and #5) was detected by flow cytometry. Specific apoptosis was calculated as described in the methods section. CalcuSyn software was used to determine the IC_{50} and generate the dose-effect curve of MLN0128 and rapamycin for each sample. Clinical information on primary AML is included in the Supplementary Table S1, combination apoptosis set B.

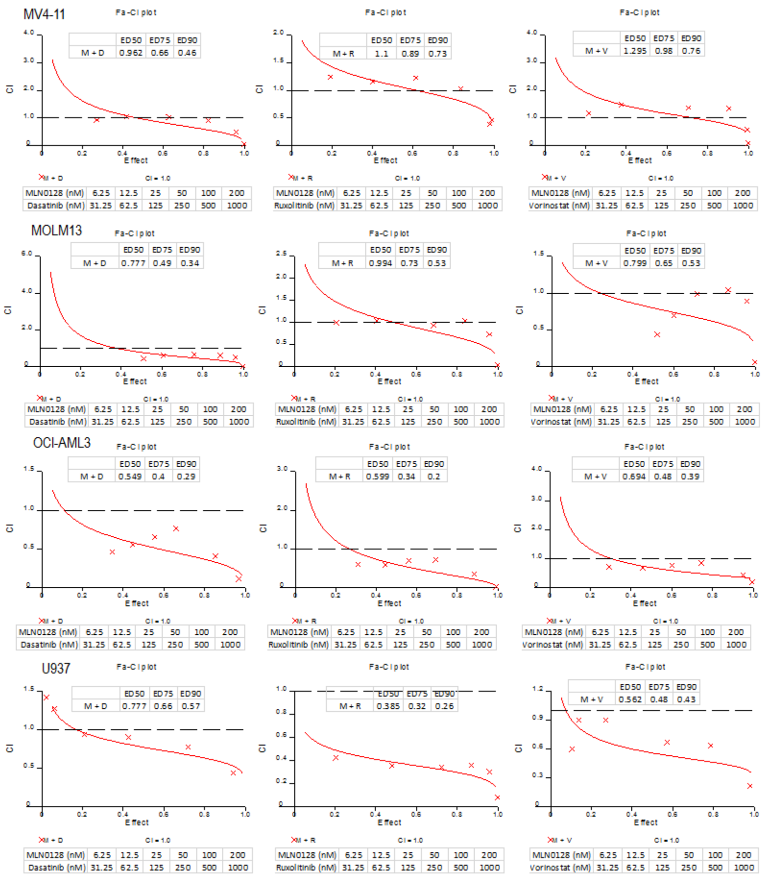

Supplementary Figure S6: Anti-leukemic effect of dual pathway inhibition in AML, related to Figure 7A. AML cell lines were treated with single and combined inhibitors at the indicated concentrations for 72 hours. Combination index and combination index plot for each treatment was generated using Calcusyn software.M: MLN0128 D: dasatinib; R: ruxolitinib; V: vorinostat.

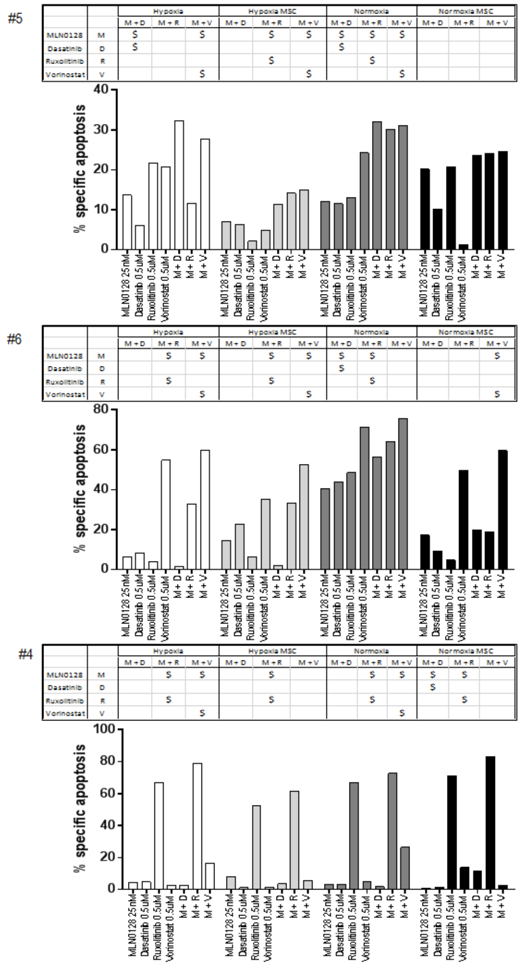

Supplementary Figure S7: Anti-leukemic efficacy of dual pathway inhibition in AML under conditions mimicking the leukemic microenvironment, related to Figure 8. Primary AML samples were cultured with or without MSC and treated with MLN0128 (25 nM), dasatinib, ruxolitinib and vorinostat (500 nM) alone and in combination under hypoxia or normoxia for 48 hours. Treatment-induced apoptosis in primary progenitor cells was detected by flow cytometry. Specific apoptosis was calculated as described in the Materials and Methods. $: Combination-treatment increased apoptosis more than 5% when compared to single agent used alone. MSC: mesenchymal stromal cells. M: MLN0128; D: dasatinib; R: ruxolitinib; V: vorinostat. Clinical information on primary AML is included in the Supplementary Table S1, combination apoptosis set B.

Supplementary Table S1: Clinical information of primary samples used in this study

	UPN	Blast	Source	Diagnosis	Disease status	FAB	Sex	molecular diagnosis mutation	CG karyotype
**Apoptosis**									
#1	4277066	77	PB	AML	Rel	M2	F	FLT3-ITD	Complex
#2	4272402	56	PB	AML	ND	M1	M	FLT3-ITD, FLT3-D835, NPM1	trisomy 4
#3	4037386	88	BM	AML	ND		M		Diploid
#4	4279490	53	PB	AML	Rel	M1	F	FLT3-ITD	inv(12)(p11.2p13)
#5	4280184	36	BM	AML	ND	M1	M		Complex
**RPPA**									
#1	3738244	69	BM	AML	Rel	M2	M	RAS (Q61K)	Diploid
#2	3727400	97	PB	AML	Rel	M1	M		t(1;10)(q25;p11.2)
#3	3741402	63	BM	AMML	ND		M	NPM1	del(9)(q13q22)
#4	3616754	87	BM	AML	Rel		M	RAS (N12 GGT-GAT)	Diploid
#5	3709076	88	BM	AML	Rel		M		Diploid
#6	3741784	90	BM	AML	Rel		M	FLT3-ITD, NPM1	Diploid
#7	3741784	90	PB	AML	Rel				trisomy 8
#8	3716342	50	PB	AML	Rel	M2	F		Complex
#9	3740978	83	BM	AML	Rel		F		Complex
#10	3719866	88	BM	AMML	Rel		M	RAS (N61 CAA-CGA)	t(11,17;12)(q23,q24;p13)
#11	2858966	80	BM	AML	Rel	RAEB-T	M		Diploid
#12	3732328	50	BM	AML	Rel	M2	M	JAK2 (V617F)	t(4;12)(q21;p11.2)
#13	3746088	52	BM	AML	ND	M2	F	FLT3-D835, NPM1	Diploid
#14	3745360	80	PB	AMOL	ND		M	FLT3-ITD, RAS (G12S)	t(6;11), complex
#15	3718532	74	PB	AML	Rel		F		t(3;21), complex
#16	3747822	65	PB	AML	ND	M0	M	RAS (two N12)	Diploid
#17	3747422	74	PB	AML	ND	M2	F	FLT3-ITD, NPM1, RAS(G12D)	Diploid
#18	3713098	96	PB	AML	Rel		F	RAS(G12D)	t(7;11), complex

(*Continued*)

	UPN	Blast	Source	Diagnosis	Disease status	FAB	Sex	molecular diagnosis mutation	CG karyotype
#19	3666302	64	PB	AML	Rel		M		Diploid
#20	3751002	60	PB	AML	ND	M2	M	CBFb-MYH11, FLT3-D835	Inv 16
#21	3750998	70	PB	AML	ND	M1	F		Diploid
#22	3715024	55	PB	AML	Rel		M	FLT3-ITD	Diploid
#23	3751374	70	BM	AMOL	ND		M	NPM1, RAS (G12D)	Diploid
#24	3749844	90	BM	AML	Rel		F	FLT3-ITD, NPM1	t(4;11;15) (q21;q13;q25)
**CyTOF**									
#1	3540878	83	PB	AML	Rel		F	FLT3-ITD, NPM1	Diploid
#2	3798414	91	PB	AML	Rel		M	RAS (N12 GGT-GCT)	DEL(12) (P11.2P13)
#3	3873322	67	PB	AML	Rel		M		Diploid
#4	3705512	83	PB	AML	Rel/Ref	M5	F	FLT3-ITD, NPM1	Diploid
#5	3705658	90	PB	AML	Rel/Ref	M2	F	FLT3-ITD	Diploid
**Western blot and apoptosis**									
#1	3615740	95	PB	AML	ND		F		Diploid
**Combination apoptosis set A**									
#1	4277066	77	PB	AML	ND	M2	M	RUNX1-RUNX1T1	t(8:21)
#2	4035774	74	PB	AML	ND	M1	F	FLT3-ITD	Diploid
#3	4038554	77	PB	AML	Rel		M		Complex
#4	4104240	89	PB	Bipheno	Rel		M		t(9;22) (q34;q11.2)
#5	4073736	83	BM	AML	Rel/Ref		F	NPM1, FLT3 ITD and CEBPA	
#6	4041728	95	PB	AMML	ND		M		Complex
#7	4049848	97	PB	AML	Rel		M		t(6;11), complex
#8	4149804	92	BM	AML	Rel/Ref		M	p53	Complex
#9	4099272	90	PB	AML	Rel/Ref		M		Complex
#10	4111142	83	PB	AML	Rel/Ref	M2	M	RUNX1, NRAS and EZH2	trisomy 8
**Combination apoptosis set B**									
#1	4340976	92	PB	AML	Ref		F	FLT3-ITD, FLT3-D835	t(X;X) (q22;q26)
#2	4378892	74	BM	AML	Ref	M5	M		Complex

(*Continued*)

	UPN	Blast	Source	Diagnosis	Disease status	FAB	Sex	molecular diagnosis mutation	CG karyotype
#3	4064236	97	PB	AML	Ref		M		Complex
#4	4522842	51	PB	AML	Ref	M4	F	FLT3-D835, RAS K: (G12V)	Diploid
#5	4095764	48	PB	AML	Ref		M	JAK2 (V617F)	Complex
#6	4522324	55	BM	AML	Ref		M	RAS, K(G12D), N (G12D+S,G13D)	Diploid

CG Karyotype: Cytogenetic Karyotype; ND: newly diagnosed; Rel: relapsed; Ref: refractory; CBFb-MYH11: fusion genes of CBFb and YMH11.

Supplementary Table S2: Antibodies used in RPPA

See Supplementary File 1

Supplementary Table S3: Antibodies used in CyTOF

Antibody	Source	Catalog #
CD45	DVS-Sunnyvale/Fluidigm	3154001B
CD38	BioLegend	303502
CD33	DVS-Sunnyvale/Fluidigm	3158001B
CD34	eBioscience	14-0349-82
CD117	DVS-Sunnyvale/Fluidigm	3143001B
CD123	DVS-Sunnyvale/Fluidigm	3151001B
CD133	Miltenyi	130-090-851
CD47	BioLegend	323102
CD99	BD Biosciences	555687
CD44	DVS-Sunnyvale/Fluidigm	3166001B
CD49d	DVS-Sunnyvale/Fluidigm	3141004B
CXCR4	DVS-Sunnyvale/Fluidigm	3175001B
p-AKT Ser473	BD Biosciences	560397
P-mTOR Ser2448	Cell signaling technology	5536BF
p-S6 Ser235/236	DVS-Sunnyvale/Fluidigm	3172008A
p-4EBP1Thr37/46	Cell signaling technology	2855BF
p-ERK Thr202/Tyr204	DVS-Sunnyvale/Fluidigm	3167005A
p-Stat3 Tyr705	Cell signaling technology	4113BF
p-STAT5 Tyr694	DVS-Sunnyvale/Fluidigm	3150005A

Underline indicates antibodies detecting the intracellular molecules

Supplementary Table S4: List of proteins significantly altered by MLN0128

See Supplementary File 2

Supplementary Table S5: Anti-leukemic efficacy of single agents in AML cell lines and primary AML samples

A.

IC50 (nM)	MV4-11	MOLM13	OCI-AML3	U937
MLN0128	21.9	20.6	31.9	75.0
Dasatinib	269.0	195.5	1690.2	3336.2
Ruxolitinib	211.2	322.2	834.1	371.2
Vorinostat	114.9	197.0	333.0	1498.5

B.

IC50 (nM)	#1	#2	#3	#4	#5
MLN0128	96.9	23.9	63.8	154.5	935.2
Dasatinib	185.5	387.7	343	3598	> 10000
Ruxolitinib	473.3	1604.7	258.5	38.5	> 10000
Vorinostat	311.0	114.0	347.2	1914.2	494.0

AML cells were treated with single agent at the indicated concentration for 72 hours. Growth inhibition in cell lines was measured by Cell titer-Glo® luminescent cell viability assay. Apoptosis induction of primary progenitor cells was detected by flow cytometry. Specific apoptosis was calculated as indicated under Methods. The IC_{50} of single agents on growth inhibition of cell lines (A) and on apoptosis induction of primary progenitor cells (B) was calculated by CalcuSyn. (Related to Figure 7 A and B)
