# Supplementary material for: MLN0128, a novel mTOR kinase inhibitor, disrupts survival signaling and triggers apoptosis in AML and AML stem/ progenitor cells
Source: Oncotarget. 2016 Jul 4;7(34):55083–97. doi: 10.18632/oncotarget.10397 (PMC5342403; doi:10.18632/oncotarget.10397)
Supplement: Supplementary file 2 [file oncotarget-07-55083-s002.docx]

**Table S2. Antibodies used in RPPA**

| Antibody | Vender | Catalog # |
| --- | --- | --- |
| AKT | Cell signaling technology | 9272 |
| p-AKT Ser473 | Cell signaling technology | 9271 |
| p-AKT Thr308 | Cell signaling technology | 2975 |
| AKT1 | Cell signaling technology | 2967 |
| AKT2 | Cell signaling technology | 2962 |
| AKT3 | Cell signaling technology | 4059 |
| AMPK.alpha | Cell signaling technology | 2532 |
| p-AMPK.alpha Thr172 | Cell signaling technology | 2535 |
| ARC | Imgenex | IMG-170 |
| ATF3 | Abcam | ab87213 |
| BAD | Cell signaling technology | 9292 |
| p-BAD.Ser112 | Cell signaling technology | 9291 |
| p-BAD Ser136 | Cell signaling technology | 9295 |
| p-BAD Ser155 | Cell signaling technology | 9297 |
| BAK | Cell signaling technology | 3792 |
| BAX | Cell signaling technology | 2772 |
| BCL2 | DAKO | M0887 |
| BCL.XL | Cell signaling technology | 2762 |
| Beclin | Cell signaling technology | 3738 |
| BID | Cell signaling technology | 2002 |
| BIM | Epitomics | 1036-1 |
| Catenin.alpha | CalBiochem | CA1030 |
| Catenin.beta | Cell signaling technology | 9562 |
| p-Catenin.beta Ser33/37/Thr41 | Cell signaling technology | 9561 |
| Cavelin.1 | Cell signaling technology | 3238 |
| CD11A | BD Transduction Lab | 610826 |
| CD20 | Epitomics | 1632-1 |
| CD31 | DAKO | M0823 |
| CD34 | Epitomics | 2150-1 |
| CD49b | BD Transduction Lab | 611016 |
| CDC2 | calbiochem | CC01 |
| CDK2 | Santa cruz biotechnology | sc-6248 |
| CDK4 | Cell signaling technology | 2906 |
| CK2.alpha | Cell signaling technology | 2656 |
| Cox.2 | Epitomics | 2169-1 |
| CREB | Epitomics | 1496-1 |
| p-CREB Ser133 | Epitomics | 1113-1 |
| Cyclin.B1 | Santa cruz biotechnology | sc-245 |
| Cyclin.D1 | Santa cruz biotechnology | sc-718 |
| Cyclin.D3 | Cell signaling technology | 2936 |
| Cyclin.E | Santa cruz biotechnology | sc-247 |
| DJ 1 | From Dr. Tek Mak |  |
| p-EGFR Tyr992 | Cell signaling technology | 2235 |
| EGFR | Santa cruz biotechnology | sc-03 |
| Egln | Millipore | 05-1327 |
| p-EIF2.alpha.Ser51 | Cell signaling technology | 9721 |
| EIF2.alpha | Cell signaling technology | 9722 |
| EIF4e | Cell signaling technology | 9742 |
| p-Elk Ser383 | Cell signaling technology | 9181 |

| ERG1.2.3 | Santa cruz biotechnology | sc-353 |
| --- | --- | --- |
| p-ERK1/2 Thr202/Tyr204 | Cell signaling technology | 9101 |
| ERK2 | Santa cruz biotechnology | sc-154 |
| FAK | Cell signaling technology | 3285 |
| Fibronectin | Epitomics | 1574-1 |
| p-FoxO1 (Thr24)/FoxO3a (Thr32) | Cell signaling technology | 9464 |
| FoxO.3alpha | Cell signaling technology | 9467 |
| p-FoxO.3alpha Ser318/321 | Cell signaling technology | 9465 |
| Gab2 | Cell signaling technology | 3239 |
| p-Gab2 Tyr452 | Cell signaling technology | 3882 |
| Galectin3 | Santa cruz biotechnology | sc-32790 |
| GAPDH | Ambion | AM4300 |
| GATA3 | BD Biosciences | 558686 |
| p-GSK3.alpha.beta.Ser21/9 | Cell signaling technology | 9331 |
| GSK3.alpha.beta | Santa cruz biotechnology | sc-7291 |
| HDAC3 | Cell signaling technology | 2632 |
| HER2 | Cell signaling technology | 2242 |
| p-HER2 Thy1248 | Upstate | 06-229 |
| HIF1.alpha | BD pharMingen | 610959 |
| hnRNPK | Santa cruz biotechnology | sc-28380 |
| HSP27 | Cell signaling technology | 2402 |
| HSP70 | Cell signaling technology | 4872 |
| HSP90 | Cell signaling technology | 4874 |
| Intergin.beta3 | Cell signaling technology | 4702 |
| p-IRS Ser1101 | Cell signaling technology | 2385 |
| JMJD6 | Abcam | ab50720 |
| JNK1 | Santa cruz biotechnology | sc-474 |
| JNK2 | Cell signaling technology | 4672 |
| p-Jun.C Ser73 | Cell signaling technology | 9164 |
| JunB | Cell signaling technology | 3755 |
| c-Kit | Epitomics | 1522-1 |
| LCK | Cell signaling technology | 2752 |
| LEF1 | Cell signaling technology | 2230 |
| LKB1 | Cell signaling technology | 3050 |
| Lyn | Cell signaling technology | 2732 |
| MCL1 | BD pharmingen | 559027 |
| MDM2 | Santa cruz biotechnology | sc-813 |
| MEK | Cell signaling technology | 9122 |
| p-MEK1/2 Ser217/221 | Cell signaling technology | 9121 |
| MSI2 | chemicon | MAB10085 |
| mTOR | Cell signaling technology | 2983 |
| p-mTOR Ser2448 | Cell signaling technology | 2971 |
| c-Myc | Cell signaling technology | 9402 |
| NF.KB.p65 | Cell signaling technology | 3034 |
| Notch1.cleaved | Cell signaling technology | 4147 |
| Notch3 | Santa cruz biotechnology | sc-5593 |
| NPM | Cell signaling technology | 3542 |
| NRP1 | Santa cruz biotechnology | 5307 |
| Nur77 | Imgenex | IMG-528 |
| OPN | Santa cruz biotechnology | sc-21742 |

| P21 | Cell signaling technology | 2946 |
| --- | --- | --- |
| p-P27 Ser10 | Epitomic | 2187-1 |
| p27 | Santa cruz biotechnology | sc-528 |
| P38 | Cell signaling technology | 9212 |
| p-p38 MAPK Thr180/Tyr182 | Cell signaling technology | 9211 |
| P53 | BD Biosciences | 554294 |
| p-P53 Ser15 | Cell signaling technology | 9284 |
| P62 | Santa cruz biotechnology | sc-28359 |
| P70S6K | Cell signaling technology | 9202 |
| p-P70S6K Thr389 | Cell signaling technology | 9205 |
| PARP | Cell signaling technology | 9542 |
| PDK1 | Cell signaling technology | 3062 |
| p-PDK1 Ser241 | Cell signaling technology | 3061 |
| PP2A | Santa cruz biotechnology | sc-18330 |
| PPAR.gamma | Santa cruz biotechnology | sc-7273 |
| PRAS40 | Invitrogen | AHO1031 |
| p-PRAS40 Thr246 | Cell signaling technology | 2997 |
| PTEN | Cell signaling technology | 9552 |
| RAC1.2.3. | Cell signaling technology | 2465 |
| S6RP | Cell signaling technology | 2217 |
| p-S6RP Ser235/236 | Cell signaling technology | 2211 |
| p-S6RP Ser240/244 | Cell signaling technology | 2215 |
| SHIP1 | Santa cruz biotechnology | sc-8425 |
| SIRT1 | Abcam | ab32441 |
| SMAC | Cell signaling technology | 2954 |
| SMAD1 | Epitomic | 1649-1 |
| SMAD4 | Santa cruz biotechnology | sc-7966 |
| SMAD6 | Cell signaling technology | 9519 |
| SRC | Upstate | 05-184 |
| p-SrcTyr416 | Cell signaling technology | 2101 |
| p-Src Tyr527 | Cell signaling technology | 2105 |
| Stat1 | Cell signaling technology | 9172 |
| p-Stat1Tyr701 | Cell signaling technology | 9171 |
| Stat3 | Upstate | 06-596 |
| p-Stat3 Tyr705 | Cell signaling technology | 9131 |
| p-Stat3 Ser727 | Cell signaling technology | 9134 |
| Stat5 | Cell signaling technology | 9352 |
| p-Stat5 Tyr694 | Cell signaling technology | 9351 |
| Stathmin | Epitomic | 1972-1 |
| Survivin | Cell signaling technology | 2802 |
| TCF4 | Santa cruz biotechnology | sc-8632 |
| TG2 | Abcam | ab2386 |
| TSC2 | Epitomic | 1613-1 |
| VEGFR2 | Cell signaling technology | 2479 |
| VHL | Novus | NB100-485 |
| 14.3.3.epsilon | Santa cruz biotechnology | sc-23957 |
| 14.3.3. sigma | Upstate | 05-632 |
| 14.3.3.zeta | chemicon | AB9746 |
| 4EBP1 | Cell signaling technology | 9452 |
| p-4EBP1 Thr37/46 | Cell signaling technology | 9459 |
| p-4EBP1 Thr70 | Cell signaling technology | 9455 |
| XIAP | Cell signaling technology | 2042 |
