# Supplementary material for: MLN0128, a novel mTOR kinase inhibitor, disrupts survival signaling and triggers apoptosis in AML and AML stem/ progenitor cells
Source: Oncotarget. 2016 Jul 4;7(34):55083–97. doi: 10.18632/oncotarget.10397 (PMC5342403; doi:10.18632/oncotarget.10397)
Supplement: Supplementary file 3 [file oncotarget-07-55083-s003.docx]

**Table S4. List of proteins significantly altered by MLN0128**

| **n = 34** | **Control** | **MLN0128** | **P < = 0.05** |
| --- | --- | --- | --- |
| 4EBP1.pT37.46 | 1.46 | -1.31 | 0 |
| 4EBP1.pT70 | -0.05 | -1.16 | 0 |
| S6RP.pS235.236 | 0.21 | -0.71 | 0.0008 |
| S6RP.pS240.244 | 0.7 | -0.75 | 0 |
| P70S6K | -0.99 | -0.61 | 0.0282 |
| FOXO3a.pS253 | 0.13 | -0.19 | 0.0361 |
| PRAS40.pT246 | -2.19 | -2.64 | 0.0145 |
| FAK | -0.96 | -1.66 | 0.0045 |
| TG2 | 1.36 | -0.36 | 0.0028 |
| CATENIN.beta | -0.24 | -1.05 | 0.0245 |
| SMAC | 1.63 | 2.05 | 0.0001 |
| PDK1.pS241 | 1.71 | 2.05 | 0.0323 |
| PARP | 1.92 | 2.57 | 0.0403 |
| CREB | 1.22 | 1.71 | 0 |
| EIF2alpha | -0.39 | 0 | 0.0016 |
| HDAC3 | 1.35 | 1.64 | 0.0012 |
| ERG1.2.3 | 0.91 | 1.58 | 0.0232 |
| NPM | 0.12 | 0.54 | 0.0014 |
| SIRT1 | -0.88 | -0.5 | 0.0095 |
| OPN | 0.02 | 0.37 | 0.0247 |
| EGFR | -1.49 | -1.13 | 0.0085 |
| HER2.pT1248 | -1.52 | -0.87 | 0 |
| HSP27 | -0.38 | 0.02 | 0.0002 |
| HSP70 | -0.15 | 0.18 | 0.0062 |
| MSI2 | -1.25 | -0.85 | 0.0013 |
| P38 | 1.69 | 2.07 | 0.0109 |
| SRC.pT416 | -0.4 | 0.02 | 0.0007 |
| STAT5 | 1.45 | 1.84 | 0.0013 |
| EGLN | -0.76 | -0.39 | 0.0001 |
| HIF1.alpha | -0.36 | -0.02 | 0.002 |
| 14.3.3 sigma | -0.09 | 0.28 | 0.0428 |
| 14.3.3 epsilon | -1.33 | -1.73 | 0.0341 |
| BAD.pS112 | 0.59 | 0.22 | 0.0013 |
| BAD.pS136 | 2.08 | 2.47 | 0.0486 |

The Tukey test identified 34 proteins that were significantly modified by MLN0128 inhibition. Left column: identified proteins; middle two columns: the mean protein intensity of control and treated groups (MLN0128) of each identified protein. All samples were normalized and transformed to a log2 scale. Color gradient represents a protein expression level (high in red, low in blue). Color intensity reflects the expression level difference. Right column: p value with a cut-off ≤ 0.05.
